# End of the Century pCO_2_ Levels Do Not Impact Calcification in Mediterranean Cold-Water Corals

**DOI:** 10.1371/journal.pone.0062655

**Published:** 2013-04-30

**Authors:** Cornelia Maier, Alexander Schubert, Maria M. Berzunza Sànchez, Markus G. Weinbauer, Pierre Watremez, Jean-Pierre Gattuso

**Affiliations:** 1 Centre National de la Recherche Scientifique-Institut National des Sciences de l’Univers, Laboratoire d’Océanographie de Villefranche, Villefranche-sur-mer, France; 2 Université Pierre et Marie Curie-Paris 6, Observatoire Océanologique de Villefranche, Villefranche-sur-mer, France; 3 Agence des Aires Marines Protegées, Brest, France; Heriot-Watt University, United Kingdom

## Abstract

Ocean acidification caused by anthropogenic uptake of CO_2_ is perceived to be a major threat to calcifying organisms. Cold-water corals were thought to be strongly affected by a decrease in ocean pH due to their abundance in deep and cold waters which, in contrast to tropical coral reef waters, will soon become corrosive to calcium carbonate. Calcification rates of two Mediterranean cold-water coral species, *Lophelia pertusa* and *Madrepora oculata*, were measured under variable partial pressure of CO_2_ (pCO_2_) that ranged between 380 µatm for present-day conditions and 930 µatm for the end of the century. The present study addressed both short- and long-term responses by repeatedly determining calcification rates on the same specimens over a period of 9 months. Besides studying the direct, short-term response to elevated pCO_2_ levels, the study aimed to elucidate the potential for acclimation of calcification of cold-water corals to ocean acidification. Net calcification of both species was unaffected by the levels of pCO_2_ investigated and revealed no short-term shock and, therefore, no long-term acclimation in calcification to changes in the carbonate chemistry. There was an effect of time during repeated experiments with increasing net calcification rates for both species, however, as this pattern was found in all treatments, there is no indication that acclimation of calcification to ocean acidification occurred. The use of controls (initial and ambient net calcification rates) indicated that this increase was not caused by acclimation in calcification response to higher pCO_2_. An extrapolation of these data suggests that calcification of these two cold-water corals will not be affected by the pCO_2_ level projected at the end of the century.

## Introduction

Ocean acidification is one of the major threats to the marine environment and has become a central research focus in marine science during the last decade. The ocean and atmosphere exchange carbon dioxide (CO_2_) [1], and the net uptake of CO_2_ by the ocean causes the pH to decline. Due to anthropogenic activity, the oocean pH has already declined by 0.1 units since pre-industrial times and will further decline by about 0.4 units until the end of the century [2]. The concentration of CO_2_ in seawater is steadily increasing at an unprecedented rate of change and it is anticipated that atmospheric CO_2_ concentrations increase four-fold between 1750 and 2100, reaching values above 1000 ppm [3]. Many calcifying organisms will be affected by ocean acidification with a decrease of the growth of their shells or skeletons [4]–[7]. Also, reproduction and larval growth are thought to be impeded [8]–[10]. For tropical coral reefs, the predictions as to the detrimental effects of ocean acidification are almost unison and foresee a decline in reef growth [11]–[15] and a shift in species composition with a decrease in diversity [16], [17]. On the other hand, it has been shown, that the response to ocean acidification can be highly variable for different taxonomic groups [18]. Investigations on cold-water corals or deep-sea corals are scarce. So far only three experimental studies on the effects of ocean acidification have been published. The rate of net calcification of *Lophelia pertusa* from Norwegian waters was shown to decline under future pCO_2_ conditions [19], [20], while no or even a positive long-term (6 months) response of net calcification was observed [20]. A third study investigated the short-term response of net calcification of the Mediterranean coral *Madrepora oculata* under pCO_2_ values reflecting past, pre-industrial and future conditions. No effect on net calcification was found between ambient and elevated pCO_2_, while calcification rates were twice as high under pre-industrial pCO_2_ than under ambient or elevated pCO_2._ This suggests that present-day calcification rates of *M. oculata* have already drastically declined since pre-industrial times [21]. Cold-water corals were initially thought to become affected by ocean acidification before their tropical relatives because they inhabit deeper and colder waters where the aragonite saturation state (Ω_a_) is lower than in shallower, warmer regions [22]. More than 70% of cold-water coral communities are found in regions that will be undersaturated with respect to aragonite by the end of the century [23]. However, experimental as well as observational evidence has shown that some cold-water corals are able to cope and maintain positive skeletal growth even in waters undersaturated in Ω_a_ [19], [20], [24]. Furthermore, the study of Form & Riebesell [20] found a decline of net calcification at elevated pCO_2_ in a short-term experiment and a stimulating effect in a long-term experiment from which they concluded that the cold-water coral *L. pertusa* is able to acclimate to higher pCO_2_. In the present study, the question of acclimation is addressed using a thorough experimental design in which net calcification of individual coral samples is repeatedly measured as a function of exposure time to respective pCO_2_ levels. The two branching cold-water coral species present in the Mediterranean Sea, *L. pertusa* and *M. oculata* are investigated to discern whether one of the two species is more resistant than the other or has a higher potential for acclimation. In the Mediterranean Sea, *M. oculata* is more widespread than *L. pertusa* [25]. This could indicate that the prevailing conditions in the Mediterranean Sea are more favourable for *M. oculata* than for *L. pertusa* and this might result in a divergent species response to changing ocean pH. It is well known, that the distribution of cold-water corals is controlled by temperature within a range of 4 to 12°C [26]. In the Mediterranean Sea, the temperature at depths where cold-water corals occur ranges between 12.5 to almost 14°C [25], [27] which is, therefore, above the common temperature range. Additionally, total alkalinity is higher in the Mediterranean Sea than open oceans and consequently absorbs more atmospheric CO_2_. It has been shown, that anthropogenic CO_2_ has already affected the Mediterranean Sea and that the pH has decreased by 0.05 to 0.14 pH units, depending on the depth considered, since pre-industrial times [28].

## Materials and Methods

The present study aimed at elucidating (1) the effect of an increase in CO_2_ (ocean acidification) on rates of net calcification on the two Mediterranean cold-water coral species, *M. oculata* and *L. pertusa*, (2) to find out whether there is a species effect and (3) to evaluate short- (shock) and longer-term (acclimation) responses in calcification. This has been addressed by measuring net calcification before, immediately after and 1, 2, 3 and 9 months after pCO_2_ has been adjusted.

### Sampling and Experimental Set-up

During the MedSeaCan cruise in June 2009, the cold-water corals *L. pertusa* and *M. oculata* were sampled in the canyon of Lacaze Duthiers using a remotely operated vehicle (ROV) and the vessel MINIBEX (COMEX, France). Corals were sampled at water depths of 500 (42°32.98'N, 03°25.21'E), 267 (42°34.98'N, 03°24.15'E) and 260 m (42°35.07'N, 03°24.14'E). On board, corals were maintained in a plastic container (1040×640×515 mm) at a controlled temperature of 12.5±0.5°C using a chilling unit attached to a water pump (1000 l h^−1^). Corals were transported back to the laboratory and maintained in a climate room at 13°C until the experiments started in August 2009. Coral branches were sub-divided into smaller fragments (Table 1) and placed into vials of 4.5 or 8 cm inner diameter and a volume of ca 300 and 1000 ml. The vials were placed in 4 aquaria that served as water baths (13°C) as well as overflow basins for seawater from the vials. Each vial received running seawater (Mediterranean surface water with a salinity of 38) and air using silicone tubings of 0.5/2.5 and 1.0/3.0 mm inner/outer diameter, respectively. The seawater was filtered through 2 layers of micron bags (5 and 1 µm) into two 100 l storage tanks in a climate room which was set to 11°C. Temperature in the water baths containing the vials was maintained at ±0.1°C using electronic temperature controllers (Corema) and heaters (Tetratec® HT75). Temperature homogeneity was obtained by circulation pumps (JBL Pro Flow 500, 500 l h^−1^). Seawater was distributed by gravity from the storage tanks to the vials at a flow rate of 32±14 ml h^−1^. Air was supplied through a small tube (ca. 8 cm height×0.7/1.0 cm inner/outer diameter) inserted vertically in the vials, preventing the bubbles to be in direct contact with the coral fragments and providing an airlift and mixing. The air was pre-mixed using mass flow controllers (MFCs, ANALYT MC-GFC17, 0–10 l for air and 0–10 ml for pure CO_2_) and an air compressor (Jun-Air OF302-25B) at a flow rate of 4×1 l min^−1^ distributed to 4×21 vials. Corals were fed 3 times a week with freshly hatched *Artemia* larvae and 1 time a week with frozen krill. The water bath containing the vials and overflowing seawater was also adjusted to the target pCO_2_ by bubbling with an air stone (HOBBY ceramic air diffuser, 150 mm). Prior to each feeding the seawater of the water bath with respective pCO_2_ was filtered (Tetratec EX 1200, 1200 l h^−1^) and the strong water flow generated by the filtration unit was used to clean vials and remove old prey and detritus.

**Table 1:** Initial size (polyp number and skeletal weight) and initial net calcification rates (G) at T_0_ (before manipulating pCO_2_) of the corals *L. pertusa* (LP) and *M. oculata* (MO) used in repeated incubations of pCO_2_ treatment A–D.

			number of polyps		skeletal weight [g]		G [% d^−1^]	
pCO_2_	Species	N	mean±S.D	Range	mean±S.D	Range	mean±S.D	Range
A	LP	4	15.75±8.96	7–28	10.22±3.23	7.11–14.73	0.005±0.003	0.000–0.008
B	LP	4	15.75±11.30	5–31	7.88±8.87	1.80–20.94	0.012±0.009	0.005–0.025
C	LP	5	20.60±14.84	7–46	8.82±7.72	3.49–22.31	0.004±0.004	0.000–0.010
D	LP	5	15.00±7.38	7–25	10.14±7.68	1.55–20.47	0.004±0.005	0.001–0.012
A-D	LP	18	16.89±10.35	5–46	9.29±6.67	1.55–22.31	0.006±0.006	0.000–0.025
A	MO	4	90.50±80.32	17–185	7.88±8.69	1.33–19.87	0.017±0.004	0.013–0.021
B	MO	7	36.14±19.28	18–77	3.21±3.44	0.72–10.25	0.034±0.033	−0.001–0.093
C	MO	6	38.00±24.27	17–83	2.88±1.57	1.28–5.67	0.014±0.015	−0.014–0.030
D	MO	6	61.17±50.06	14–132	8.26±10.61	0.59–24.41	0.024±0.015	0.007–0.052
A-D	MO	23	52.61±45.94	14–185	5.25±6.80	0.59–24.41	0.023±0.022	−0.014–0.093

Means ± S.D.

### Determination of Rates of Net Calcification

Before changing the pCO_2_ levels in the experimental set-up, net calcification for each coral fragments was determined using the alkalinity anomaly technique [29] in order to provide an initial control (T_0_) at ambient pCO_2_. Subsequently, the CO_2_ concentration of the air used to bubble the vials was adjusted to 4 pCO_2_ treatment levels (A–D) with treatment A at 280 (low), B at 400 (ambient), C at 700 (elevated) and D at 1000 ppm (elevated) using the MFCs. To adjust to lower pCO2 than ambient (treatment A, 280 ppm), soda lime was used to generate low-CO2-air (5–10 ppm) which was mixed with pure CO2. For treatment B ambient air was used and for treatment C (700 ppm) and D (1000 ppm) ambient air was mixed with pure CO_2._ The exact mixing of air or CO_2_-free air with pure CO2 was adjusted by a LI-COR CO2-analyser (LI-6252). The pH in the different treatments during coral maintenance was monitored on a weekly base using a commercial pH module and electrode (IKS aquastar) which was calibrated to the NBS standard (buffers 4 and 7). This was done to control that treatment levels remained relatively constant, however, these measurements were not used to assess the carbonate chemistry, which was determined by analysis of A_T_ and C_T_ (see below for details) 1) during 2-day incubation to determine calcification rates via the alkalinity anomaly technique and 2) in maintenance vials containing corals and in blanks (vials without corals) after 9 months when net calcification rates were established using the buoyant weight technique.

To discriminate between short- and longer-term effects, net calcification was determined immediately after changing the pCO_2_ by transferring coral fragments directly from ambient into incubation vials adjusted to target air-CO_2_ mix (T_1_) and at about monthly intervals during the first 3 months (T_2_, T_3_ and T_4_, with 29, 57 and 89 days of exposure, respectively) using the alkalinity anomaly technique. Additionally, net calcification was again determined after approximately 9 months (267±14 days) with the buoyant weight technique [30] using a balance (Mettler Toledo) with a precision of 1 mg.

For determination of net calcification rates using the alkalinity anomaly technique, corals and blanks (seawater without corals) were placed for 2 days in the same type of vials than those in which they were maintained but with a constant volume of 200 or 700 ml. To maintain pCO_2_ levels constant vials were aerated with the same air-CO_2_ mix that was also used during maintenance periods for treatments A–D. At the end of the incubation, seawater was sub-sampled to determine inorganic nutrients, dissolved inorganic carbon (C_T_) and total alkalinity (A_T_) as described in Maier et al. [21]. Other parameters of the carbonate chemistry (pCO_2_, pH on the total scale, pH_T_, and Ω_a_) were determined from C_T_, A_T_, temperature (13°C), salinity (38) and hydrostatic pressure (0) using the software package seacarb [32] (Table 2 and S1). Rates of net calcification were calculated from differences in A_T_ from blanks (seawater without corals incubated in parallel to coral samples) and coral incubations and were corrected for changes in the concentration of inorganic nutrients. For the incubation at T_3_, no samples for inorganic nutrient concentrations were taken. Therefore, the regression functions between calcification rates corrected (or uncorrected) for inorganic nutrient release (Figure S1) were used to make the nutrient correction for T_3_. Data were normalized to the initial skeletal dry weight of coral fragments and reported in % d^−1^ [21], [31] using the exponential growth function G [% d^−1^] = ((W_n_/W_0_)^1/n^ −1) * 100; with G = net calcification rate, W_n_ = Weight after n days, W_0_ = initial weight and d time interval (days) for growth increment (alkalinity anomaly method = 2 days, buoyant weight method = 267 days).

**Table 2:** Parameters of the carbonate chemistry for the total anomaly technique at T_0_ (ambient air, prior to adjusting to respective pCO_2_ levels) and at T_1_-T_4_ (immediately and 1, 2 and 3 months after adjusting pCO_2_, respectively).

T	pCO_2_	Coral	A_T_	C_T_	pH_T_	PCO_2_	Ω_a_	PO_4_	NH_4_
treatment			[µmol kg^−1^]	[µmol kg^−1^]		[µatm]		[µmolkg-1]	[µmol kg-1]
0	all	blank	**2616**±**27**	**2374**±**21**	8.05±0.03	447±32	2.7±0.16	0.03±0.07	1.03±1.48
1–4	A	blank	**2606**±**5**	**2313**±**3**	8.14±0.01	349±6	3.2±0.04	0.11±0.12	3.93±0.31
1–4	B	blank	**2598**±**15**	**2370**±**10**	8.03±0.02	468±26	2.6±0.12	0.02±0.02	1.50±0.34
1–4	C	blank	**2598**±**1**	**2438**±**5**	7.88±0.02	688±27	1.9±0.07	0.03±0.02	0.77±0.63
1–4	D	blank	**2602**±**6**	**2492**±**4**	7.76±0.01	929±25	1.5±0.03	0.03±0.04	0.77±0.90
0	all	LP	**2571**±**32**	**2356**±**41**	8.00±0.07	507±93	2.4±0.30	0.51±0.34	4.84±5.17
1–4	A	LP	**2483**±**54**	**2236**±**23**	8.10±0.02	379±10	2.8±0.11	0.89±0.13	3.73±0.85
1–4	B	LP	**2529**±**14**	**2315**±**11**	8.00±0.01	489±13	2.4±0.06	0.79±0.09	4.02±0.40
1–4	C	LP	**2495**±**62**	**2352**±**56**	7.85±0.01	713±19	1.7±0.05	0.83±0.05	5.23±0.66
1–4	D	LP	**2477**±**36**	**2387**±**31**	7.73±0.02	969±25	1.3±0.08	1.26±0.08	5.44±0.63
0	all	MO	**2546**±**47**	**2333**±**39**	8.00±0.06	499±74	2.4±0.28	0.44±0.49	7.83±4.97
1–4	A	MO	**2525**±**7**	**2256**±**11**	8.10±0.02	380±12	2.9±0.11	0.78±0.07	9.13±3.39
1–4	B	MO	**2528**±**19**	**2320**±**12**	8.00±0.04	495±50	2.3±0.21	0.65±0.05	5.32±0.28
1–4	C	MO	**2523**±**4**	**2375**±**6**	7.86±0.02	707±52	1.8±0.06	0.73±0.04	6.25±0.54
1–4	D	MO	**2506**±**12**	**2408**±**7**	7.74±0.02	947±20	1.4±0.06	1.06±0.13	8.76±3.08

Blank is seawater incubated in parallel without coral (N≥4, see Table S1) and used to subtract from A_T_ of coral incubations to determine net calcification rates. LP is *L. pertusa*, MO is *M. oculata*. A_T_ and C_T_ (bold) were measured after 2-day incubations and other parameters were calculated from A_T_, C_T_, temperature of 13°C and salinity of 38 using the software package seacarb [31]. Values are means ± S.D. (N is given in Table 1).

### Statistical Analysis

Statistical analyses were conducted using the software package Statistica 7.0. A repeated measures analysis of variance (ANOVA) was used. Only coral fragments for which net calcification rates were established at all time steps (T_0_–T_4_ and buoyant weight after 9 months) were considered for analysis as this is a pre-requisite for repeated measures testing. Therefore N is the same for all repeated measurements (Table S1). For independent samples (non-repeated measures analyses) a one-way ANOVA comparison of carbonate chemistry between pCO_2_ treatments or initial size and initial net calcification rates (T_0_) was used. If applicable, a post-hoc test was performed using the Honest Significance Difference (HSD) test for either equal or unequal N. The respective statistical tests used are also given in the text, tables and figure legends. Values are given as mean ± S.D. unless stated otherwise.

## Results and Discussion

### Initial Carbonate Chemistry, Concentration of Inorganic Nutrients and Calcification

For the initial incubation at ambient pCO_2_ (T_0_), the average A_T_ and C_T_ of blank controls (incubation without corals) was 2616±27 and 2374±21 µmol kg^−1^ (± standard deviation, throughout this paper; N = 30). pH_T_, pCO_2_ and Ω_a_ were 8.05±0.03, 447.3±31.7 µatm and 2.68±0.16, respectively. The concentrations of phosphate and ammonium were 0.03±0.07 and 1.03±1.48 µmol kg^−1^ (N = 16). A_T_ and C_T_ decreased during the 2-day incubations of both coral species; A_T_ was on average 2546±47 and 2571±32 µmol kg^−1^ and C_T_ was 2333±39 and 2356±41 µmol kg^−1^ (N = 23 and 18), respectively. As a consequence, pH_T_ and Ω_a_ were also lower in coral vials than in blanks while pCO_2_ increased (Table 2). For both species, pH_T_ was lower by 0.05 units and Ω_a_ was lower by 0.03, whereas pCO_2_ increased by 52 for *M. oculata* and 60 µatm for *L. pertusa*. Cold-water corals can release significant amounts of inorganic nutrients [33], and phosphate concentrations increased by 0.413 and 0.483 µmol kg^−1^ to 0.44±0.49 and 0.51±0.34 µmol kg^−1^, while ammonium increased by 6.80 and 3.81 µmol kg^−1^ to 7.83±4.97 and 4.84±5.17 µmol kg^−1^ for *M. oculata* and *L. pertusa* (N = 18 and 22), respectively.

Before changing the pCO_2_ to respective treatment levels A–D (low-high pCO_2_), mean calcification rates were 0.006±0.006% d^−1^ for *L. pertusa* (N = 18) and 0.023±0.022% d^−1^ for *M. oculata* (N = 23) (Table 1). For both species, the rates of net calcification were significantly correlated with skeletal weight. Smaller coral fragments exhibit higher net calcification rates following a negative logarithmic trend (Figure S2). This negative dependence of size and age for cold-water coral calcification is in accordance with earlier studies [19], [21], [36]. However, despite the fact that we worked with a relatively large size range, neither size (weight or polyp number), nor initial calcification rates measured under ambient pCO_2_ (Table 1) differed significantly between the coral fragments used in the four pCO_2_ treatments (1-way ANOVA, p≥0.15) and net calcification rates were well within the range of earlier findings [19]–[21], [34], [35].

### Carbonate Chemistry of Blanks after Adjusting pCO_2_ Levels

After the CO_2_ was adjusted to the 4 pCO_2_ levels (A–D), the carbonate chemistry changed accordingly (Table 2). For the blanks, A_T_ was relatively uniform around 2600 µmol kg^−1^ for treatments A to D. The C_T_ of blanks increased from 2313±3 to 2492±4 µmol kg^−1^, and pH_T_ decreased from 8.14±0.01 to 7.76±0.01, Ω_a_ from 3.2±0.04 to 1.5±0.03 and pCO_2_ levels ranged from 349±6 to 929±25 µatm for treatments A to D, respectively. For time step T_1_–T_4_, A_T_ was not significantly different between treatments A–D (One-way ANOVA, p>0.2), while it was significant between maintenance vials buoyant weight, treatment B and D, and buoyant weight, treatment D and T_4_ treatment D. Other parameters of the carbonate chemistry (C_T_, pCO_2_, pH and Ω_a_) were in general significantly different between single pCO_2_ treatments (One-way ANOVA, Tukey Honest-Significant Difference (HSD) post-hoc test, p<0.05) with exceptions for adjacent treatment levels where p-values between treatments were >0.05. The actual pCO_2_ values of seawater differed from those applied by the MFCs and revealed a reduced range between 349 to 929 instead of 280 to 1000 µatm, respectively. This is probably due to the fact that all 4 pCO_2_ treatments were maintained and incubated in the same climate room and mixing of seawater with the overlying air most likely took place due to the vertical water circulation that was generated by the aeration system.

### Carbonate Chemistry and Calcification Rates in Coral Incubation after Adjusting pCO_2_ Levels

For coral incubation of T_1_–T_4_, the A_T_ of seawater containing *L. pertusa* was on average 2496±45, while A_T_ for *M. oculata* incubation was on average 2520±21 (Table 2 and Table S1). The C_T_ of *L. pertusa* increased from treatment A–D from 2236±23 to 2492±4 µmol kg^−1^ while C_T_ of *M. oculata* increased from 2256±11 to 2408±7 µmol kg^−1^ for treatment A to D, respectively. Similar as for the T_0_, the DIC, pH_T_ and Ω_a_ were slightly lower in coral vials than in corresponding blanks while pCO_2_ was higher (Table 2). This means, that also other parameters of the carbonate system than the A_T_ changed during the 2-day incubation as a consequence of coral calcification and metabolism. It is evident, that these shifts cannot be avoided as the determination of calcification rates is based on the fact, that the precipitation of 1 mole CaCO_3_ decreases the A_T_ by 2 mole and C_T_ by 1 mole [37]. Also, the CO_2_ released by coral respiration and calcification into the surrounding seawater was apparently not completely equilibrated by aeration with respective air-CO_2_ gas mix and the pCO_2_ levels of coral vials increased by an average 20 to 40 µatm during the 2-day incubation (Table 2). The A_T_ decreased by about 100 µmole relative to blanks, and there was no significant difference of A_T_ between pCO_2_ treatments or repeated incubation T_1_ to T_4_ at the end of incubation (Table S2). The excretion of inorganic nutrients by cold-water corals increases the A_T_, i.e. it counteracts the decrease caused by calcification. Thus, calcification rates determined by the alkalinity anomaly technique are an underestimate of actual calcification rates if not corrected for. In a previous study using freshly collected cold-water corals it has been demonstrated that calcification rates of both species would be underestimated by 10% if inorganic nutrients were not taken into account [21]. In the present study, inorganic nutrient concentrations were used to correct calcification rates using the following linear equations: G_L.persusa_ = 1.025*G_uncorrected_ +000019 (R = 0.997, N = 81, p<<0.001) and G_M.oculata_ = 1.027*G_uncorrected_ +0.00093 (R = 0.993, N = 102, p<<0.001) (Figure S1). This means that the ratio of inorganic nutrient release to net calcification was 4-times higher for freshly collected corals than for corals maintained in aquaria.

### Calcification Rates and pCO_2_ Treatment Effects

**Figure 1:**
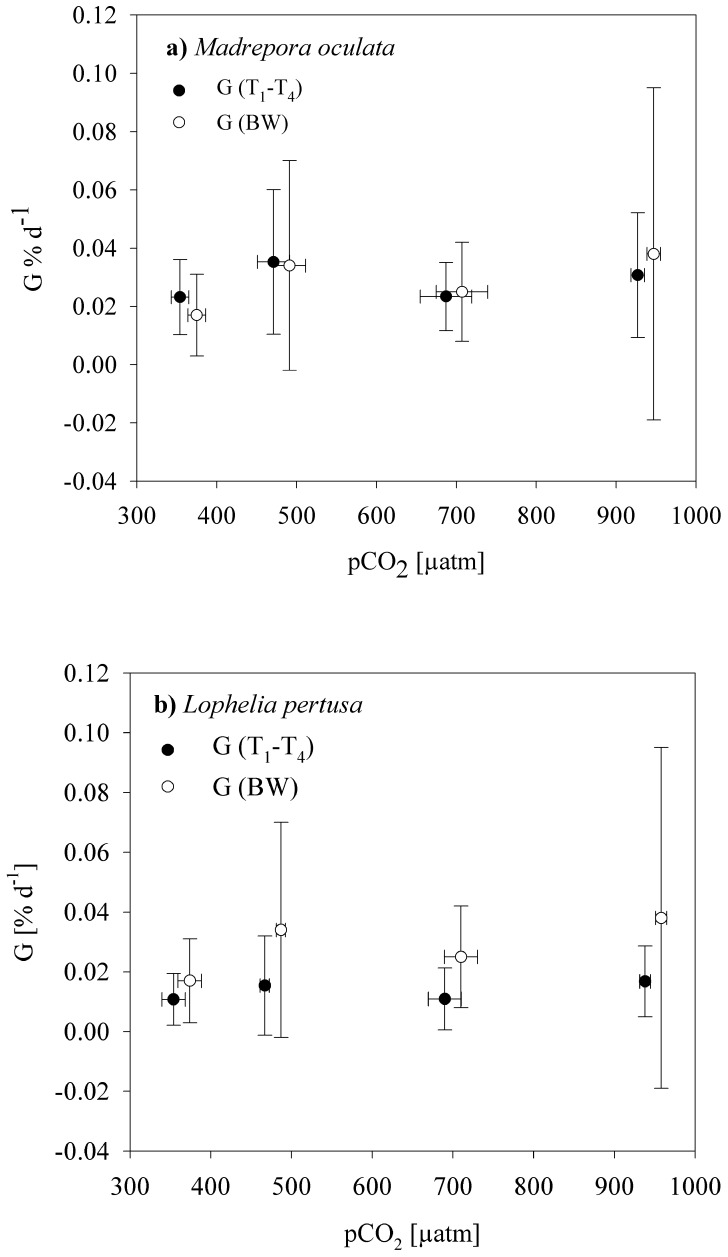
Net calcification rates of a) ***M.*** **oculata**** **and b)** ***L. pertusa*** **at various pCO_2_ levels pooled over T_1_–T_4_ (total anomaly technique) and from buoyant weight technique spanning 4 or 9 months, respectively.** **** Values are mean ± SD, N≥4 (Table 1).

After the pCO_2_ had been adjusted to the intended treatment levels, calcification rates were measured immediately (T_1_) and 1, 2 and 3 months (T_2_–T_4_) after adjusting the pCO_2_ levels using the alkalinity anomaly technique and additionally after 9 months using the buoyant weighing technique. Pooled data from repeated measurements (average T_1_–T_4_) of alkalinity anomaly corresponding to approximately 3-months of coral growth and the buoyant weight after 9-months provided similar results and average calcification rates of *L. pertusa* slightly varied between treatments A and D from 0.011±0.008 to 0.017±0.012% d^−1^ for the alkalinity anomaly method and between 0.010±0.008 and 0.021±0.037% d^−1^ for the buoyant weight technique (Figure 1, Table S3). For *M. oculata* average values ranged between 0.023±0.012 and 0.035±0.025% d^−1^ and between 0.017±0.014 and 0.038±0.057% d^−1^ for the alkalinity anomaly and buoyant weight technique, respectively. For both coral species, there was neither a significant effect between methods used to measure calcification rates (time span of maintenance at respective pCO_2_ levels) nor a significant pCO_2_ treatment effect or a combined effect of methods (exposure time) and pCO_2_ levels (Repeated measures ANOVA, p>0.05, Table S4A). As we used two different methods to establish net calcification rates over mid-term and longer-term calcification, we provide a methods comparison for a similar time interval (mid-term) between alkalinity anomaly and buoyant weight showing that the 2 methods provide similar results (SI 1). These data were not included in the manuscript, as they do not comprise the same N, which is mandatory for the repeated measures design used here.

### Short-term (Shock) and Longer-term (Acclimation) Response to Variable pCO_2_

**Figure 2:**
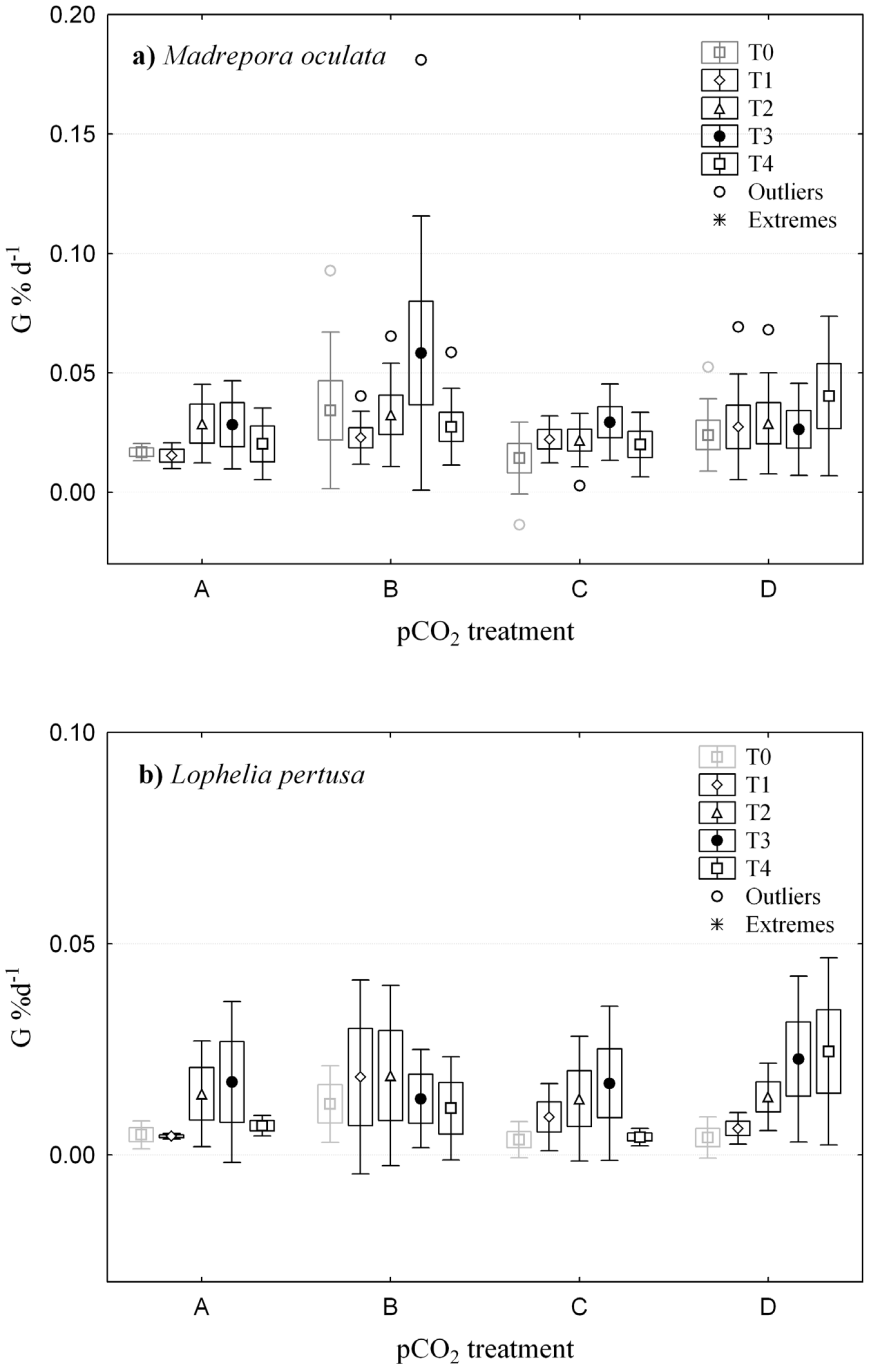
Net calcification rates (G) of a) ***M.*** **oculata**** **and b)** ***L. pertusa*** **at T_0_–T4 at the 4 pCO_2_ treatment levels (pCO_2_ values at different time steps and for the two species are given in Table S1).** **** The pCO_2_ at T_0_ was at ambient pCO_2_ and served as initial control, while T_1_–T_4_ was after pCO_2_ has been changed to respective treatment levels. Values are mean ± SE and SD.

The experimental approach used in the present study was designed to discriminate between short-term “shock” effects due to fast changes in pCO_2_ levels and longer-term acclimation response of net calcification. The results from repeated determination of calcification rates over time revealed no short-term, or long-term response in calcification to higher pCO_2_ for either of the two species investigated (Figure 2). There was a continuous increase in calcification rates for the coral *L. pertusa* in treatment D which had highest pCO_2_ levels (Figure 1), however this increase was statistically not significant (repeated measures ANOVA, Table S4B). Thus, our data do not support an earlier suggestion for a potential short-term shock response with long-term acclimation of net calcification rates as proposed by Form & Riebesell [20]. Yet, there was an effect of time on net calcification rates for both coral species. In general, average calcification rates (pooled for pCO_2_ treatments) increased until T_3_ and then decreased at T_4_ again (repeated measures ANOVA, unequal N HSD, Table S4C). This pattern was independent of pCO_2_ treatment and similar for *L. pertusa* and *M. oculata*. This indicates, that factors other than pCO_2_ must have been responsible for the changes in calcification rates with time, that were either driven by intrinsic controls (e.g. reproductive cycles) or a general acclimation to the maintenance conditions in the vials or aquarium (independent of pCO_2_).

The lack of pCO_2_ treatment effects seems to contradict other studies reporting on a short-term calcification response of *L. pertusa* to elevated pCO_2_ levels [19], [20]. In the following, an attempt is made to reconcile these contradictory findings. Form & Riebesell found a negative correlation between calcification rates and CO_2_ concentration in their short-term experiment. However, this negative correlation might have been due to one very high value for calcification rates at the lowest pCO_2_ which forced the slope to a negative trend while all other values were within a certain range independent of pCO_2_ ([20]; Figure 2) similar to the findings of the present study. In the short-term study by Maier et al. [19] there was also a clear negative response to increasing pCO_2_ on net calcification rates. In that study, the experimental set up was different as very small incubation vials (50 ml) and a closed system approach was used. Thus, the initial values for carbonate chemistry were comparable with the present study and that of Form & Riebesell [20], however, the carbonate chemistry changed drastically during incubation and mean pCO_2_ values as high as 2160 µatm were reached ([19]; Table S4). The conclusion with respect to an expected 50% decrease in calcification rates by the end of the century might thus have been a misinterpretation with respect to relating net calcification rates to the initial pCO_2_ range and not to the much higher pCO_2_ values that were actually reached during incubation due to the closed system approach and small incubation volume.

For *M. oculata*, so far only one study exists with respect to ocean acidification and it addresses the short-term pCO_2_ effect on net calcification rates [21]. In that study, the pCO_2_ was changed in two ways: 1) to that of pre-industrial concentrations (285 ppm) and 2) to values projected for the end of the century (865 ppm). Similar to the present study, there was no change in net calcification rates between ambient and future pCO_2_ levels, while calcification rates doubled when pCO_2_ was set to values of pre-industrial times indicating that the increase in pCO_2_ that took place since pre-industrial times had already a negative impact on *M. oculata* calcification.

The results of the present study are in contrast to the acclimation hypothesis postulated by Form & Riebesell [20]. Their conclusion with respect to acclimation was based on the fact, that there was a negative short-term response, while in the long-term study higher pCO_2_ had even higher calcification rates than the two lower pCO_2_ levels. However, there is indication that their data can be interpreted differently. First, the long-term experiment of Form & Riebesell [20] lacked the ambient pCO_2_ control and did thus not cover the same pCO_2_ range than their short-term study. However, an ambient pCO_2_ treatment in the long-term study would have been pivotal, specifically because the negative response in the short-term experiment was caused by a higher calcification rate at ambient pCO2. Second, the study by Form & Riebesell [20] also lacked initial controls. It is therefore possible, that the coral fragments used in the high pCO_2_ treatment had already higher initial calcification rates under ambient pCO_2_ conditions. In contrast, the experimental design of the present study comprised both initial and ambient controls and a time-series for calcification rates which allowed a better evaluation of shock or short-term responses versus long-term acclimation in calcification rates. Due to a lack in short-term response of calcification for the range in pCO_2_ levels studied, our data do not provide evidence that acclimation had played a role in the long-term calcification response to increasing pCO_2_. This does not mean that no acclimation took place, it rather means that the mechanism(s) enabling cold-water corals to maintain calcification rates constant over a large pCO_2_ range (independent of the duration of the exposure) remain to be identified.

### Summary View of pCO2 Effects on Cold-water Coral Calcification

For a pCO_2_ range between 350 and 1000 µatm, no effect on net calcification rates, neither for short- nor long-term exposure could be distinguished, and there is evidence that the recently postulated cold-water coral acclimation hypothesis [20] does not hold as such. The present study revealed significant changes observed as function of time for all pCO_2_ treatments, but this must be attributed to other causes than pCO_2_ either related to aquarium conditions or coral biology. Including the previous 3 studies on cold-water coral response to ocean acidification and the results of the present study a certain pattern emerges: the response of cold-water corals *L. pertusa* and *M. oculata* to increasing pCO_2_ is non-linear and net calcification rates remain constant for a pCO_2_ range between ambient and somewhere above 1000 µatm where Ω_a_ is already close to or even below 1. The negative short-term response in the study by Maier et al. (2009) indicated, that once a threshold at high pCO_2_ has been reached, a significant decline in net calcification rates can be expected with increasing pCO_2_ it can further be assumed that this threshold lies somewhere below 2000 µatm. For the Mediterranean coral *M. oculata* it appears that a 1^st^ threshold had already been surpassed since pre-industrial as indicated by the increase in calcification rates of *M. oculata* at reduced pCO_2_ [21]. In this respect, the exceptionally high calcification value of *L. pertusa* at lowest pCO_2_ in the short-term experiment of Form & Riebesell [20] might be indicative of such a threshold between present-day and pre-industrial pCO_2_, however, this needs further investigation.

A non-linear response is contrary to findings for tropical, zooxanthellate corals, that generally reveal a linear, negative calcification response to increasing pCO_2_ [13]–[15]. However, a non-linear response had already been revealed for temperate zooxanthellate corals which, similar to the Mediterranean cold-water corals, remained unaffected within a large range of present-day to end of the century projections of pCO_2_ [18], [38], [39] and a drastic reduction in calcification at a pCO_2_ of 2850 µatm [40]. The responses of the organisms to increasing ocean acidification are variable and complex [18] and even enhanced calcification at higher pCO_2_ had been proposed for some taxa [41]–[43].

Up to now it is unclear how the corals are able to resist increasing pCO_2_ levels and how they maintain calcification rates constant over such a large pCO_2_ gradient. It has been proposed that cold-water corals are able to resist increasing ocean acidification by their ability to maintain a high pH within their calicoblastic, calcifying fluid [44]. The way the calcifying fluid is sheltered and replenished with cations from ambient seawater is crucial in how a coral responds to increasing ocean acidification [45]–[46]. Also, an explanation why temperate corals can resist to higher pCO_2_ levels are their lower growth rates [38] as less carbonate ions will be required in the same time for calcification. This could also explain why cold-water corals grow in waters with an Ω_a_ around 1, while fast growing tropical corals are found in waters with an Ω_a_ above 3.5. Overall, cold-water corals seem well adapted to low Ω_a_ which may explain their resistance to increasing pCO_2_ levels up to a certain threshold. Tropical corals can experience drastic short-term changes of pCO_2_ in the close environment due to diurnal changes in CO_2_ uptake and release driven by light-dependent changes in the metabolic functioning of reef organisms [47]. Nothing is known on naturally occurring short-term changes in the seawater carbonate chemistry close to cold-water corals but due to the lack of photosynthetic activity in these depths they are likely less pronounced than in tropical reef systems. Nevertheless the question remains if any naturally occurring short-term changes render cold-water corals resistant to fast changes in pCO_2_ and a large range of pCO_2_ with values reaching more than 1000 µatm. Finally, it is still questionable if acclimation of calcification to increasing pCO_2_ is a likely scenario in the natural environment. The resistance to increasing pCO_2_ levels and the maintenance of constant calcification requires energy and might thus be sustainable during short-term exposure while energy requirements might not be sustained over longer time of exposure to higher pCO_2_. This might especially be the case in the natural cold-water coral environment, where regular food supply as usually provided during aquarium maintenance is not always guaranteed and where other stressors such as predation, disease and temperature abnormalities may further impede coral growth.

## Supporting Information

Figure S1 **Calcification rates (G) of** ***M. oculata*** **and** ***L. pertusa*** **corrected and uncorrected for inorganic nutrient reslease during incubation.** Correlation analysis were significant with R = 0.993, N = 102, p<<0.001 and R = 0.997, N = 81, p<<0.001 for *M. oculata* and *L. pertusa,* respectively.
(PDF)

Figure S2 **Calcification rates (G) versus skeleletal weight (SW) of** ***M. oculata*** **and** ***L. pertusa*** **under ambient pCO_2_ conditions (T_0_).** Logarithmic regressions are significant with p = 0.01 for both species; N = 23 and 18 for *M. oculata* and *L. pertusa*, respectively.
(PDF)

Table S1 **Parameters of the carbonate chemistry (CC) and the inorganic nutrients (IN) phosphate (PO_4_) and ammonium (NH_4_) for the incubation times T_0_–T_4_ and pCO_2_ treatments A–D after 2-day incubation using the alkalinity anomaly technique.** Additionally, CC was established after 9 months (267 days) when net calcification rates were measured using the buoyant weight (BW) technique. T_0_ was established prior to adjusting pCO_2_ at ambient for all treatments, while T_1_–T_4_ and BW are values derived from 2-day incubations immediately and 1, 2 and 3 months after adjusting pCO_2_ and after 9 months, respectively. LP (*Lophelia pertusa),* MO *(Madrepora oculata)* and blank (incubated in parallel without coral) at respective pCO_2_ treatment levels. Values are given as mean ± S.D.
(PDF)

Table S2 **Post-hoc results (Tukey honest-significance difference test for unequal N) of breakdown ANOVA for parameters of the carbonate chemistry (A_T_, C_T_, pCO_2_, pH_T_, Ω_a_) for the coral incubations with T_1_–T_4_ (directly and 1, 2, 3 months after pCO_2_ was changed to respective treatment levels) established during 2-day incubation using alkalinity anomaly and after 9 months (267 days) using buoyant weight (BW) technique to determine net calcification rates.** Matrix of p-values for *L. pertusa* at upper right and for *M. oculata* at lower left part of the table with p<0.05 (italic). Bold values are corresponding treatments at different incubation times.
(PDF)

Table S3 **Average calcification rates (G) of the cold-water corals** ***Lophelia pertusa*** **(LP) and** ***Madrepora oculata*** **(MO) at T_0_ to T_4_ determined by the total anomaly technique in monthly time intervals and 2 days of incubation, and calcification rates determined by buoyant weight (G(BW)) after maintenance of corals for 9–10 months under respective pCO_2_ treatment levels.**
(PDF)

Table S4 **Statistical results for repeated measures ANOVA of calcification rates (G).** **A** Comparison between total alkalinity (TA) method (average G, pooled T1–T4) and Buoyant Weighting (BW) for pCO_2_ treatments A–D; and **B** for comparison of repeated measurements T_0_–T_4_ for the 4 pCO_2_ treatments. Table **C** gives the matrix for p-values of the Tukey-Honest-Significance post-hoc comparison for unequal N of the variable R1 (T_0_–T_4_) for *M. oculata* (lower left) and *L. pertusa (upper right).* Significant p are marked in bold, italic
(PDF)

File S1 **Method comparison between total alkalinity anomaly (TAA) and buoyant weight (BW) technique to establish net calcification rates (G) of** ***M. oculata*** **and** ***L. pertusa*** **.**
(PDF)
