# Supplementary material for: End of the Century pCO2 Levels Do Not Impact Calcification in Mediterranean Cold-Water Corals
Source: PLoS One. 2013 Apr 30;8(4):e62655. doi: 10.1371/journal.pone.0062655 (PMC3640017; doi:10.1371/journal.pone.0062655)
Supplement: Figure S1 — Calcification rates (G) of M. oculata and L. pertusa corrected and uncorrected for inorganic nutrient reslease during incubation. Correlation analysis were significant with R = 0.993, N = 102, p<<0.001 and R = 0.997, N = 81, p<<0.001 for M. oculata and L. pertusa, respectively. (PDF) [file pone.0062655.s001.pdf]

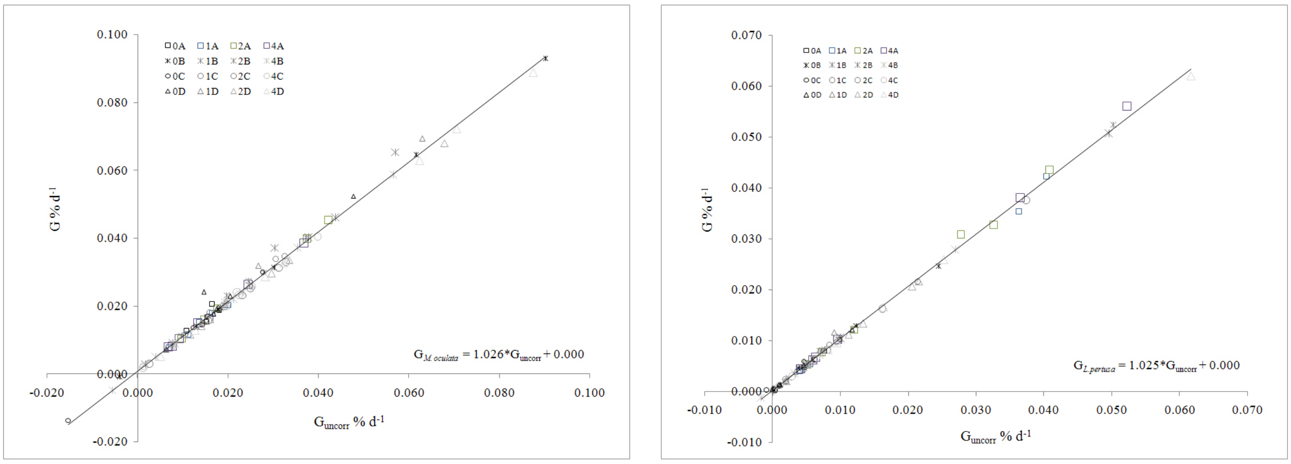

Figure S1 Calcification rates (G) of *M. oculata* and *L. pertusa* corrected and uncorrected for inorganic nutrient reslease during incubation. Correlation analysis were significant with R = 0.993, N = 102 , p<< 0.001 and R = 0.997, N = 81, p <<0.001 for *M. oculata* and *L. pertusa,* respectively.
