# Supplementary material for: End of the Century pCO2 Levels Do Not Impact Calcification in Mediterranean Cold-Water Corals
Source: PLoS One. 2013 Apr 30;8(4):e62655. doi: 10.1371/journal.pone.0062655 (PMC3640017; doi:10.1371/journal.pone.0062655)
Supplement: Figure S2 — Calcification rates (G) versus skeleletal weight (SW) of M. oculata and L. pertusa under ambient pCO2 conditions (T0). Logarithmic regressions are significant with p = 0.01 for both species; N = 23 and 18 for M. oculata and L. pertusa, respectively. (PDF) [file pone.0062655.s002.pdf]

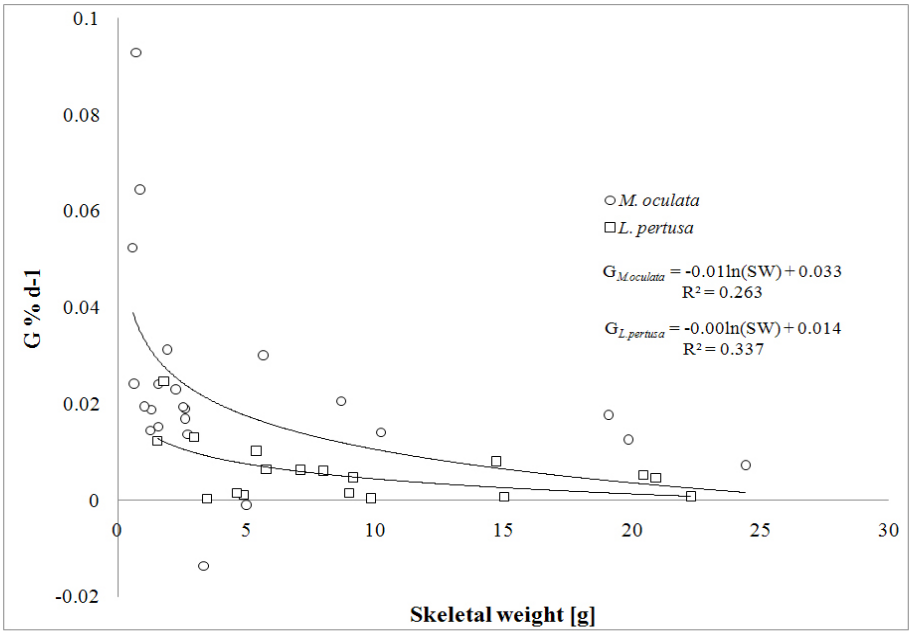

Figure S2 Calcification rates (G) versus skeleletal weight (SW) of *M. oculata* and *L. pertusa* under ambient pCO_2 conditions (T_0). Logarithmic regressions are significant with $p = 0.01$ for both species; N= 23 and 18 for *M. oculata* and *L. pertusa*, respectively.
