# Supplementary material for: End of the Century pCO2 Levels Do Not Impact Calcification in Mediterranean Cold-Water Corals
Source: PLoS One. 2013 Apr 30;8(4):e62655. doi: 10.1371/journal.pone.0062655 (PMC3640017; doi:10.1371/journal.pone.0062655)
Supplement: Table S1 — Parameters of the carbonate chemistry (CC) and the inorganic nutrients (IN) phosphate (PO4) and ammonium (NH4) for the incubation times T0–T4 and pCO2 treatments A–D after 2-day incubation using the alkalinity anomaly technique. Additionally, CC was established after 9 months (267 days) when net calcification rates were measured using the buoyant weight (BW) technique. T0 was established prior to adjusting pCO2 at ambient for all treatments, while T1–T4 and BW are values derived from 2-day incubations immediately and 1, 2 and 3 months after adjusting pCO2 and after 9 months, respectively. LP (Lophelia pertusa), MO (Madrepora oculata) and blank (incubated in parallel without coral) at respective pCO2 treatment levels. Values are given as mean ± S.D. (PDF) [file pone.0062655.s003.pdf]

Table S1 Parameters of the carbonate chemistry (CC) and the inorganic nutrients (IN) phosphate (PO_4) and ammonium (NH_4) for the incubation times T_0-T_4 and pCO_2 treatments A-D after 2-day incubation using the alkalinity anomaly technique. Additionally, CC was established after 9 months (267 days) when net calcification rates were measured using the buoyant weight (BW) technique. T_0 was established prior to adjusting pCO_2 at ambient for all treatments, while T_1-T_4 and BW are values derived from 2-day incubations immediately and 1, 2 and 3 months after adjusting pCO_2 and after 9 months, respectively. LP (*Lophelia pertusa),* MO *(Madrepora oculata)* and blank (incubated in parallel without coral) at respective pCO_2 treatment levels. Values are given as mean ± S.D.

t	pCO_2 level	Coral	N_C	A_T [µmol kg^{-1}]	C_T [µmol kg^{-1}]	pH_T	pCO_2 [µatm]	Ω_a	N_{IN}	PO_4 [µmol kg^{-1}]	NH_4 [µmol kg^{-1}]
0	A	blank	7	**2621 ± 12**	**2377 ± 7**	8.05 ± 0.03	446 ± 33	2.7 ± 0.2	4	0.08 ± 0.15	1.74 ± 2.45
0	B	blank	7	**2639 ± 38**	**2383 ± 29**	8.07 ± 0.02	424 ± 14	2.8 ± 0.1	4	0.01 ± 0.01	0.58 ± 0.46
0	C	blank	5	**2608 ± 20**	**2369 ± 29**	8.04 ± 0.03	452 ± 38	2.6 ± 0.1	4	0.01 ± 0.00	1.40 ± 1.76
0	D	blank	7	**2602 ± 11**	**2362 ± 15**	8.05 ± 0.02	446 ± 22	2.7 ± 0.1	4	0.01 ± 0.00	0.39 ± 0.19
1	A	blank	7	**2607 ± 9**	**2313 ± 12**	8.14 ± 0.02	347 ± 20	3.2 ± 0.1	7	0.01 ± 0.00	4.14 ± 2.60
1	B	blank	8	**2604 ± 9**	**2381 ± 11**	8.01 ± 0.02	487 ± 23	2.5 ± 0.1	8	0.01 ± 0.00	0.83 ± 0.66
1	C	blank	6	**2610 ± 6**	**2444 ± 15**	7.90 ± 0.04	669 ± 75	2.0 ± 0.2	7	0.01 ± 0.00	0.31 ± 0.21
1	D	blank	7	**2611 ± 2**	**2496 ± 17**	7.77 ± 0.04	914 ± 88	1.5 ± 0.1	7	0.01 ± 0.00	0.30 ± 0.25
2	A	blank	7	**2613 ± 16**	**2298 ± 9**	8.17 ± 0.01	318 ± 10	3.4 ± 0.1	4	0.00 ± 0.00	2.68 ± 2.00
2	B	blank	8	**2601 ± 9**	**2379 ± 9**	8.01 ± 0.01	488 ± 14	2.5 ± 0.1	4	0.00 ± 0.01	0.85 ± 0.30
2	C	blank	6	**2604 ± 8**	**2440 ± 6**	7.89 ± 0.02	672 ± 36	2.0 ± 0.1	5	0.03 ± 0.02	0.33 ± 0.10
2	D	blank	7	**2600 ± 15**	**2491 ± 14**	7.76 ± 0.02	936 ± 46	1.5 ± 0.1	4	0.02 ± 0.00	0.19 ± 0.08
3	A	blank	7	**2578 ± 48**	**2284 ± 30**	8.14 ± 0.05	341 ± 38	3.2 ± 0.3	n/a		
3	B	blank	6	**2588 ± 35**	**2410 ± 120**	8.04 ± 0.09	457 ± 110	2.1 ± 0.8	n/a		
3	C	blank	6	**2583 ± 8**	**2434 ± 8**	7.86 ± 0.02	720 ± 28	1.8 ± 0.1	n/a		
3	D	blank	5	**2585 ± 14**	**2479 ± 10**	7.76 ± 0.02	945 ± 49	1.5 ± 0.1	n/a		
4	A	blank	7	**2596 ± 25**	**2332 ± 20**	8.09 ± 0.02	397 ± 16	2.9 ± 0.1	3	0.32 ± 0.22	4.97 ± 2.14
4	B	blank	8	**2619 ± 31**	**2382 ± 27**	8.04 ± 0.01	458 ± 16	2.6 ± 0.1	3	0.05 ± 0.04	2.81 ± 0.99
4	C	blank	6	**2591 ± 7**	**2428 ± 15**	7.89 ± 0.05	677 ± 77	1.9 ± 0.2	3	0.05 ± 0.04	1.68 ± 1.23
4	D	blank	7	**2565 ± 84**	**2461 ± 70**	7.75 ± 0.03	945 ± 52	1.5 ± 0.1	3	0.06 ± 0.08	1.81 ± 1.72
BW	A	blank	7	**2602 ± 56**	**2382 ± 52**	8.01 ± 0.01	495 ± 14	2.5 ± 0.1			
BW	B	blank	7	**2561 ± 26**	**2336 ± 26**	8.02 ± 0.01	469 ± 19	2.5 ± 0.1			
BW	C	blank	6	**2610 ± 69**	**2457 ± 60**	7.87 ± 0.02	719 ± 33	1.9 ± 0.1			
BW	D	blank	5	**2656 ± 81**	**2518 ± 82**	7.81 ± 0.07	848 ± 139	1.7 ± 0.3			
0	A	LP	4	**2562 ± 37**	**2358 ± 49**	7.98 ± 0.09	541 ± 130	2.3 ± 0.4	4	0.46 ± 0.16	1.31 ± 1.52
0	B	LP	4	**2571 ± 17**	**2350 ± 28**	8.01 ± 0.03	483 ± 46	2.5 ± 0.2	4	0.53 ± 0.47	3.79 ± 2.59
0	C	LP	5	**2568 ± 49**	**2357 ± 61**	7.99 ± 0.08	518 ± 123	2.4 ± 0.4	5	0.59 ± 0.36	8.44 ± 7.46
0	D	LP	5	**2580 ± 20**	**2359 ± 31**	8.01 ± 0.05	488 ± 69	2.5 ± 0.3	5	0.54 ± 0.41	5.76 ± 4.76
1	A	LP	4	**2550 ± 14**	**2275 ± 26**	8.12 ± 0.03	363 ± 29	3.0 ± 0.1	4	0.31 ± 0.19	4.07 ± 1.39
1	B	LP	4	**2522 ± 61**	**2319 ± 53**	7.98 ± 0.01	512 ± 10	2.3 ± 0.1	4	0.51 ± 0.17	5.95 ± 1.96
1	C	LP	5	**2528 ± 58**	**2377 ± 47**	7.87 ± 0.05	692 ± 71	1.8 ± 0.2	5	0.45 ± 0.36	4.40 ± 4.02
1	D	LP	5	**2554 ± 25**	**2449 ± 30**	7.75 ± 0.02	939 ± 62	1.4 ± 0.1	5	0.95 ± 0.37	6.69 ± 2.48
2	A	LP	4	**2450 ± 85**	**2193 ± 56**	8.09 ± 0.05	371 ± 38	2.7 ± 0.3	4	1.08 ± 0.40	4.96 ± 2.65
2	B	LP	4	**2511 ± 51**	**2318 ± 47**	7.96 ± 0.01	537 ± 18	2.2 ± 0.1	4	0.91 ± 0.29	4.30 ± 1.50
2	C	LP	5	**2474 ± 115**	**2334 ± 102**	7.85 ± 0.03	711 ± 26	1.7 ± 0.2	5	0.88 ± 0.26	6.67 ± 3.57
2	D	LP	5	**2465 ± 76**	**2376 ± 72**	7.72 ± 0.02	986 ± 40	1.3 ± 0.1	5	1.25 ± 0.32	6.28 ± 3.07
3	A	LP	4	**2412 ± 131**	**2197 ± 73**	8.12 ± 0.02	367 ± 37	2.9 ± 0.2	n/a		
3	B	LP	4	**2531 ± 28**	**2301 ± 28**	8.04 ± 0.03	448 ± 37	2.5 ± 0.2	n/a		
3	C	LP	5	**2424 ± 157**	**2292 ± 137**	7.83 ± 0.04	729 ± 40	1.6 ± 0.2	n/a		
3	D	LP	5	**2428 ± 114**	**2338 ± 102**	7.72 ± 0.04	969 ± 75	1.3 ± 0.2	n/a		
4	A	LP	4	**2520 ± 27**	**2277 ± 27**	8.06 ± 0.01	414 ± 16	2.6 ± 0.1	4	1.27 ± 0.42	2.16 ± 1.03
4	B	LP	4	**2552 ± 53**	**2323 ± 36**	8.03 ± 0.03	458 ± 34	2.5 ± 0.2	4	0.94 ± 0.12	1.80 ± 1.16
4	C	LP	5	**2552 ± 16**	**2405 ± 12**	7.86 ± 0.03	720 ± 49	1.8 ± 0.1	5	1.16 ± 0.30	4.62 ± 4.87
4	D	LP	5	**2462 ± 77**	**2385 ± 50**	7.72 ± 0.00	983 ± 19	1.2 ± 0.2	5	1.57 ± 0.47	3.35 ± 3.75
BW	A	LP	5	**2558 ± 23**	**2311 ± 15**	8.06 ± 0.05	423 ± 56	2.7 ± 0.3			
BW	B	LP	5	**2615 ± 100**	**2388 ± 81**	8.02 ± 0.03	483 ± 28	2.5 ± 0.2			
BW	C	LP	4	**2557 ± 9**	**2428 ± 9**	7.82 ± 0.01	799 ± 20	1.6 ± 0.0			
BW	D	LP	3	**2611 ± 80**	**2514 ± 60**	7.73 ± 0.06	1024 ± 121	1.4 ± 0.2			
0	A	MO	4	**2547 ± 35**	**2337 ± 21**	7.99 ± 0.08	510 ± 92	2.4 ± 0.4	4	0.74 ± 0.78	11.31 ± 9.86
0	B	MO	7	**2563 ± 47**	**2349 ± 43**	8.00 ± 0.04	497 ± 44	2.4 ± 0.2	7	0.22 ± 0.24	5.20 ± 1.75
0	C	MO	6	**2552 ± 44**	**2329 ± 42**	8.02 ± 0.05	474 ± 70	2.5 ± 0.3	5	0.38 ± 0.24	6.09 ± 3.04
0	D	MO	6	**2518 ± 54**	**2314 ± 42**	7.98 ± 0.08	519 ± 103	2.3 ± 0.4	6	0.56 ± 0.60	10.01 ± 2.54
1	A	MO	4	**2550 ± 18**	**2275 ± 11**	8.11 ± 0.04	365 ± 40	3.0 ± 0.3	4	0.42 ± 0.33	8.75 ± 3.17
1	B	MO	7	**2550 ± 25**	**2340 ± 23**	7.99 ± 0.02	503 ± 22	2.3 ± 0.1	7	0.31 ± 0.28	5.74 ± 2.41
1	C	MO	6	**2540 ± 34**	**2383 ± 20**	7.88 ± 0.04	671 ± 55	1.9 ± 0.2	6	0.43 ± 0.35	5.60 ± 3.74
1	D	MO	6	**2523 ± 35**	**2421 ± 34**	7.75 ± 0.02	937 ± 43	1.4 ± 0.1	6	0.81 ± 0.54	9.06 ± 1.92
2	A	MO	4	**2514 ± 19**	**2249 ± 17**	8.10 ± 0.02	370 ± 22	2.8 ± 0.1	4	0.93 ± 0.42	9.15 ± 1.28
2	B	MO	7	**2529 ± 53**	**2322 ± 46**	7.99 ± 0.02	505 ± 24	2.3 ± 0.1	7	0.79 ± 0.27	7.33 ± 2.85
2	C	MO	6	**2528 ± 39**	**2389 ± 26**	7.84 ± 0.07	754 ± 152	1.7 ± 0.3	6	0.80 ± 0.28	8.71 ± 4.13
2	D	MO	6	**2483 ± 56**	**2400 ± 39**	7.70 ± 0.07	1005 ± 91	1.3 ± 0.2	6	1.28 ± 0.53	12.77 ± 7.73
3	A	MO	4	**2501 ± 20**	**2213 ± 9**	8.12 ± 0.00	373 ± 29	2.9 ± 0.0	n/a		
3	B	MO	7	**2482 ± 63**	**2297 ± 36**	7.98 ± 0.10	518 ± 125	2.1 ± 0.5	n/a		
3	C	MO	6	**2497 ± 44**	**2360 ± 34**	7.84 ± 0.02	738 ± 35	1.7 ± 0.1	n/a		
3	D	MO	6	**2509 ± 37**	**2398 ± 27**	7.77 ± 0.04	876 ± 72	1.5 ± 0.1	n/a		
4	A	MO	4	**2537 ± 33**	**2289 ± 33**	8.07 ± 0.01	410 ± 11	2.7 ± 0.0	4	0.99 ± 0.46	9.48 ± 7.86
4	B	MO	7	**2553 ± 26**	**2323 ± 22**	8.03 ± 0.02	454 ± 26	2.5 ± 0.1	7	0.84 ± 0.20	2.88 ± 2.92
4	C	MO	6	**2528 ± 40**	**2370 ± 33**	7.88 ± 0.04	666 ± 58	1.9 ± 0.2	6	0.96 ± 0.34	4.45 ± 3.06
4	D	MO	6	**2509 ± 29**	**2413 ± 23**	7.73 ± 0.03	969 ± 72	1.4 ± 0.1	6	1.09 ± 0.30	4.44 ± 3.06
BW	A	MO	5	**2576 ± 68**	**2353 ± 79**	8.02 ± 0.03	481 ± 56	2.5 ± 0.1			
BW	B	MO	6	**2590 ± 70**	**2367 ± 60**	8.01 ± 0.02	484 ± 23	2.5 ± 0.2			
BW	C	MO	4	**2597 ± 65**	**2447 ± 46**	7.86 ± 0.05	727 ± 69	1.8 ± 0.2			
BW	D	MO	5	**2656 ± 89**	**2535 ± 82**	7.79 ± 0.03	900 ± 68	1.6 ± 0.1			
