# Supplementary material for: End of the Century pCO2 Levels Do Not Impact Calcification in Mediterranean Cold-Water Corals
Source: PLoS One. 2013 Apr 30;8(4):e62655. doi: 10.1371/journal.pone.0062655 (PMC3640017; doi:10.1371/journal.pone.0062655)
Supplement: Table S2 — Post-hoc results (Tukey honest-significance difference test for unequal N) of breakdown ANOVA for parameters of the carbonate chemistry (AT, CT, pCO2, pHT, Ωa) for the coral incubations with T1–T4 (directly and 1, 2, 3 months after pCO2 was changed to respective treatment levels) established during 2-day incubation using alkalinity anomaly and after 9 months (267 days) using buoyant weight (BW) technique to determine net calcification rates. Matrix of p-values for L. pertusa at upper right and for M. oculata at lower left part of the table with p<0.05 (italic). Bold values are corresponding treatments at different incubation times. (PDF) [file pone.0062655.s004.pdf]

Table S2 Post-hoc results (Tukey honest-significance difference test for unequal N) of breakdown ANOVA for parameters of the carbonate chemistry (A_T, C_T, pCO_2, pH_T, Ω_a) for the coral incubations with T_1-T_4 (directly and 1, 2, 3 months after pCO_2 was changed to respective treatment levels) established during 2-day incubation using alkalinity anomaly and after 9 months (267 days) using buoyant weight (BW) technique to determine net calcification rates. Matrix of p-values for *L. pertusa* at upper right and for *M. oculata* at lower left part of the table with $p < 0.05$ (italic). Bold values are corresponding treatments at different incubation times.

A_T			***Lophelia pertusa***																			
			**T1**				**T2**				**T3**				**T4**				**BW**			
			**A**	**B**	**C**	**D**	**A**	**B**	**C**	**D**	**A**	**B**	**C**	**D**	**A**	**B**	**C**	**D**	**A**	**B**	**C**	**D**
Madrepora oculata	**T1**	**A**		1.000	1.000	1.000	**0.985**	1.000	0.990	0.969	**0.761**	1.000	0.551	0.602	**1.000**	1.000	1.000	0.958	1.000	1.000	1.000	1.000
		**B**	1.000		1.000	1.000	1.000	**1.000**	1.000	1.000	0.978	**1.000**	0.903	0.927	1.000	**1.000**	1.000	1.000	1.000	1.000	1.000	0.993
		**C**	1.000	1.000		1.000	1.000	1.000	**1.000**	0.999	0.960	1.000	**0.856**	0.888	1.000	1.000	**1.000**	0.999	1.000	1.000	1.000	0.997
		**D**	1.000	1.000	1.000		0.981	1.000	0.988	**0.962**	0.737	1.000	0.523	**0.574**	1.000	1.000	1.000	**0.949**	1.000	1.000	1.000	1.000
	**T2**	**A**	**1.000**	1.000	1.000	1.000		1.000	1.000	1.000	**1.000**	1.000	1.000	1.000	**1.000**	0.977	0.985	1.000	**0.920**	0.871	0.971	0.659
		**B**	1.000	**1.000**	1.000	1.000	1.000		1.000	1.000	0.994	**1.000**	0.958	0.971	1.000	**1.000**	1.000	1.000	1.000	**1.000**	1.000	0.979
		**C**	1.000	1.000	**1.000**	1.000	1.000	1.000		1.000	1.000	1.000	**1.000**	1.000	1.000	0.984	**0.990**	1.000	0.941	0.898	**0.980**	0.697
		**D**	0.979	0.865	0.963	**0.999**	1.000	0.997	0.994		1.000	1.000	1.000	**1.000**	1.000	0.955	0.969	**1.000**	0.866	0.810	0.947	**0.585**
	**T3**	**A**	**0.985**	0.947	0.972	1.000	**1.000**	0.999	0.996	1.000		0.992	1.000	1.000	**0.977**	0.715	0.761	1.000	**0.997**	0.969	0.997	0.833
		**B**	0.976	**0.754**	0.958	0.999	1.000	**0.991**	0.992	1.000	1.000		0.952	0.967	1.000	**1.000**	1.000	1.000	1.000	**1.000**	1.000	0.981
		**C**	1.000	0.999	**1.000**	1.000	1.000	1.000	**1.000**	1.000	1.000	1.000		1.000	0.899	0.499	**0.551**	1.000	0.276	0.270	**0.481**	0.166
		**D**	1.000	0.999	1.000	**1.000**	1.000	1.000	1.000	**1.000**	1.000	1.000	1.000		0.924	0.550	0.601	**1.000**	0.320	0.308	0.530	**0.190**
	**T4**	**A**	**1.000**	1.000	1.000	1.000	**1.000**	1.000	1.000	1.000	**1.000**	1.000	1.000	1.000		1.000	1.000	0.999	**1.000**	1.000	1.000	0.993
		**B**	1.000	**1.000**	1.000	1.000	0.999	**1.000**	1.000	0.819	0.923	**0.689**	0.998	0.998	1.000		1.000	0.940	1.000	**1.000**	1.000	1.000
		**C**	1.000	1.000	**1.000**	1.000	1.000	1.000	**1.000**	1.000	1.000	1.000	**1.000**	1.000	1.000	1.000		0.957	1.000	1.000	**1.000**	1.000
		**D**	1.000	0.999	1.000	**1.000**	1.000	1.000	1.000	**1.000**	1.000	1.000	1.000	**1.000**	1.000	0.998	1.000		0.832	0.774	0.930	**0.547**
	**BW**	**A**	1.000	1.000	1.000	0.958	**0.716**	1.000	0.998	0.175	**0.199**	0.163	0.647	0.750	**0.954**	1.000	0.953	0.741		1.000	1.000	1.000
		**B**	0.983	0.994	0.993	0.591	0.370	**0.964**	0.947	0.017	0.058	**0.015**	0.309	0.246	0.730	**0.997**	0.728	0.238	1.000		1.000	1.000
		**C**	0.995	0.999	0.998	0.911	0.684	0.996	**0.985**	0.241	0.264	0.229	**0.630**	0.712	0.904	1.000	**0.904**	0.704	1.000	1.000		1.000
		**D**	0.085	0.154	0.113	0.015	0.003	0.089	0.050	**0.000**	0.000	0.000	0.002	**0.004**	0.014	0.189	0.014	**0.003**	0.632	0.892	0.991	

C_T			***Lophelia pertusa***																			
			**T1**				**T2**				**T3**				**T4**				**BW**			
			**A**	**B**	**C**	**D**	**A**	**B**	**C**	**D**	**A**	**B**	**C**	**D**	**A**	**B**	**C**	**D**	**A**	**B**	**C**	**D**
Madrepora oculata	**T1**	**A**		1.000	0.553	0.004	**0.974**	1.000	0.996	0.569	**0.991**	1.000	1.000	0.991	**1.000**	1.000	0.136	0.392	**1.000**	0.962	0.086	0.002
		**B**	0.263		0.991	0.095	0.436	**1.000**	1.000	0.993	0.629	**1.000**	1.000	1.000	1.000	**1.000**	0.744	0.963	1.000	**1.000**	0.541	0.021
		**C**	0.001	0.953		0.927	0.009	0.994	**1.000**	1.000	0.033	0.932	**0.785**	1.000	0.516	0.998	**1.000**	1.000	0.972	1.000	**1.000**	0.388
		**D**	0.000	0.062	0.996		0.000	0.107	0.246	**0.920**	0.000	0.035	0.013	**0.308**	0.004	0.153	1.000	**0.978**	0.058	0.785	1.000	**0.999**
	**T2**	**A**	**1.000**	0.011	0.000	0.000		0.405	0.200	0.009	**1.000**	0.691	0.878	0.155	**0.981**	0.311	0.001	0.004	**0.567**	0.137	0.001	0.000
		**B**	0.389	**1.000**	0.883	0.032	0.021		1.000	0.995	0.599	**1.000**	1.000	1.000	1.000	**1.000**	0.773	0.971	1.000	**1.000**	0.570	0.023
		**C**	0.001	0.946	**1.000**	0.996	0.000	0.872		1.000	0.370	1.000	**1.000**	1.000	0.994	1.000	**0.939**	0.998	1.000	1.000	**0.793**	0.053
		**D**	0.000	0.478	1.000	**1.000**	0.000	0.326	1.000		0.035	0.938	0.798	**1.000**	0.532	0.999	1.000	**1.000**	0.975	1.000	1.000	**0.377**
	**T3**	**A**	**0.776**	0.003	0.000	0.000	**0.999**	0.005	0.000	0.000		0.834	0.947	0.308	**0.994**	0.503	0.004	0.018	**0.742**	0.131	0.001	0.000
		**B**	1.000	**0.854**	0.062	0.000	0.671	**0.974**	0.058	0.002	0.175		1.000	1.000	1.000	**1.000**	0.492	0.836	1.000	**1.000**	0.324	0.009
		**C**	0.018	1.000	**1.000**	0.749	0.000	1.000	**1.000**	0.997	0.000	0.399		1.000	1.000	1.000	**0.284**	0.631	1.000	0.995	**0.180**	0.004
		**D**	0.000	0.553	1.000	**1.000**	0.000	0.391	1.000	**1.000**	0.000	0.004	0.999		0.987	1.000	0.965	**0.999**	1.000	1.000	0.848	**0.068**
	**T4**	**A**	**1.000**	0.560	0.005	0.000	**0.986**	0.709	0.005	0.001	**0.532**	1.000	0.070	0.001		0.999	0.120	0.359	**1.000**	0.952	0.077	0.001
		**B**	0.745	**1.000**	0.568	0.005	0.091	**1.000**	0.550	0.094	0.018	**0.999**	0.973	0.122	0.950		0.856	0.989	1.000	**1.000**	0.666	0.033
		**C**	0.022	1.000	**1.000**	0.717	0.000	1.000	**1.000**	0.995	0.000	0.432	**1.000**	0.998	0.080	0.980		1.000	0.617	1.000	**1.000**	0.787
		**D**	0.000	0.156	1.000	**1.000**	0.000	0.088	1.000	**1.000**	0.000	0.000	0.915	**1.000**	0.000	0.018	0.897		0.913	1.000	1.000	**0.511**
	**BW**	**A**	**0.074**	1.000	0.999	0.415	**0.002**	1.000	0.999	0.933	**0.001**	0.733	1.000	0.958	**0.222**	0.999	1.000	0.651		1.000	0.424	0.013
		**B**	0.014	**1.000**	1.000	0.668	0.000	**0.997**	1.000	0.995	0.000	**0.196**	1.000	0.998	0.054	**0.905**	1.000	0.881	1.000		0.974	0.160
		**C**	0.000	0.116	**0.917**	1.000	0.000	0.076	**0.924**	0.995	0.000	0.002	**0.535**	0.991	0.000	0.024	**0.507**	1.000	0.292	0.586		0.966
		**D**	0.000	0.000	0.000	**0.011**	0.000	0.000	0.000	**0.001**	0.000	0.000	0.000	**0.001**	0.000	0.000	0.000	**0.004**	0.000	0.000	0.395	

pCO_2			***Lophelia pertusa***																			
			**T1**				**T2**				**T3**				**T4**				**BW**			
			**A**	**B**	**C**	**D**	**A**	**B**	**C**	**D**	**A**	**B**	**C**	**D**	**A**	**B**	**C**	**D**	**A**	**B**	**C**	**D**
Madrepora oculata	**T1**	**A**		0.015	0.000	0.000	**1.000**	0.001	0.000	0.000	**1.000**	0.018	0.000	0.000	**1.000**	0.522	0.000	0.000	**0.986**	0.193	0.000	0.000
		**B**	0.024		0.000	0.000	0.030	**1.000**	0.000	0.000	0.011	**1.000**	0.000	0.000	0.338	**0.994**	0.000	0.000	0.603	**1.000**	0.000	0.000
		**C**	0.000	0.005		0.000	0.000	0.002	**1.000**	0.000	0.000	0.000	**1.000**	0.000	0.000	0.000	**1.000**	0.000	0.000	0.000	**0.361**	0.000
		**D**	0.000	0.000	0.000		0.000	0.000	0.000	**0.997**	0.000	0.000	0.000	**1.000**	0.000	0.000	0.000	**1.000**	0.000	0.000	0.050	**0.914**
	**T2**	**A**	**1.000**	0.023	0.000	0.000		0.002	0.000	0.000	**1.000**	0.035	0.000	0.000	**1.000**	0.684	0.000	0.000	**0.998**	0.293	0.000	0.000
		**B**	0.018	**1.000**	0.007	0.000	0.017		0.000	0.000	0.001	**1.000**	0.000	0.000	0.052	**0.736**	0.000	0.000	0.140	**0.999**	0.000	0.000
		**C**	0.000	0.000	**1.000**	0.000	0.000	0.000		0.000	0.000	0.000	**1.000**	0.000	0.000	0.000	**1.000**	0.000	0.000	0.000	**0.714**	0.000
		**D**	0.000	0.000	0.000	**0.989**	0.000	0.000	0.000		0.000	0.000	0.000	**1.000**	0.000	0.000	0.000	**1.000**	0.000	0.000	0.001	**1.000**
	**T3**	**A**	**1.000**	0.145	0.000	0.000	**1.000**	0.120	0.000	0.000		0.013	0.000	0.000	**0.985**	0.353	0.000	0.000	**0.911**	0.052	0.000	0.000
		**B**	0.002	**1.000**	0.049	0.000	0.002	**1.000**	0.003	0.000	0.033		0.000	0.000	0.372	**0.996**	0.000	0.000	0.642	**1.000**	0.000	0.000
		**C**	0.000	0.000	**0.961**	0.000	0.000	0.000	**1.000**	0.000	0.000	0.000		0.000	0.000	0.000	**1.000**	0.000	0.000	0.000	**0.944**	0.000
		**D**	0.000	0.000	0.000	**0.945**	0.000	0.000	0.001	**0.145**	0.000	0.000	0.014		0.000	0.000	0.000	**1.000**	0.000	0.000	0.004	**0.999**
	**T4**	**A**	**0.998**	0.559	0.000	0.000	**0.998**	0.487	0.000	0.000	**0.999**	0.148	0.000	0.000		0.998	0.000	0.000	**1.000**	0.869	0.000	0.000
		**B**	0.550	**0.986**	0.000	0.000	0.543	**0.987**	0.000	0.000	0.787	**0.757**	0.000	0.000	1.000		0.000	0.000	1.000	**1.000**	0.000	0.000
		**C**	0.000	0.007	**1.000**	0.000	0.000	0.009	**1.000**	0.000	0.000	0.061	**0.941**	0.000	0.000	0.000		0.000	0.000	0.000	**0.859**	0.000
		**D**	0.000	0.000	0.000	**1.000**	0.000	0.000	0.000	**1.000**	0.000	0.000	0.000	**0.360**	0.000	0.000	0.000		0.000	0.000	0.001	**1.000**
	**BW**	**A**	**0.128**	1.000	0.001	0.000	**0.125**	1.000	0.000	0.000	**0.378**	0.999	0.000	0.000	**0.915**	1.000	0.001	0.000		0.972	0.000	0.000
		**B**	0.110	**1.000**	0.001	0.000	0.107	**1.000**	0.000	0.000	0.348	**0.998**	0.000	0.000	0.892	**1.000**	0.001	0.000	1.000		0.000	0.000
		**C**	0.000	0.001	**0.997**	0.004	0.000	0.002	**1.000**	0.000	0.000	0.009	**1.000**	0.183	0.000	0.000	**0.996**	0.000	0.000	0.000		0.001
		**D**	0.000	0.000	0.000	**1.000**	0.000	0.000	0.001	**0.727**	0.000	0.000	0.008	**1.000**	0.000	0.000	0.000	**0.975**	0.000	0.000	0.047	

pH_T			***Lophelia pertusa***																			
			**T1**				**T2**				**T3**				**T4**				**BW**			
			**A**	**B**	**C**	**D**	**A**	**B**	**C**	**D**	**A**	**B**	**C**	**D**	**A**	**B**	**C**	**D**	**A**	**B**	**C**	**D**
Madrepora oculata	**T1**	**A**		0.000	0.000	0.000	**1.000**	0.000	0.000	0.000	**1.000**	0.001	0.000	0.000	**0.942**	0.122	0.000	0.000	**0.876**	0.033	0.000	0.000
		**B**	0.001		0.001	0.000	0.005	**1.000**	0.000	0.000	0.001	**1.000**	0.000	0.000	0.110	**0.929**	0.000	0.000	0.170	**1.000**	0.000	0.000
		**C**	0.000	0.009		0.001	0.000	0.017	**1.000**	0.000	0.000	0.000	**0.981**	0.000	0.000	0.000	**1.000**	0.000	0.000	0.000	**0.886**	0.002
		**D**	0.000	0.000	0.000		0.000	0.000	0.016	**0.995**	0.000	0.000	0.134	**0.997**	0.000	0.000	0.005	**1.000**	0.000	0.000	0.651	**1.000**
	**T2**	**A**	**1.000**	0.002	0.000	0.000		0.000	0.000	0.000	**1.000**	0.016	0.000	0.000	**1.000**	0.582	0.000	0.000	**1.000**	0.221	0.000	0.000
		**B**	0.001	**1.000**	0.014	0.000	0.001		0.001	0.000	0.000	**0.999**	0.000	0.000	0.008	**0.382**	0.003	0.000	0.014	**0.979**	0.000	0.000
		**C**	0.000	0.001	**1.000**	0.001	0.000	0.001		0.000	0.000	0.000	**1.000**	0.000	0.000	0.000	**1.000**	0.002	0.000	0.000	**0.999**	0.022
		**D**	0.000	0.000	0.000	**0.767**	0.000	0.000	0.000		0.000	0.000	0.002	**1.000**	0.000	0.000	0.000	**1.000**	0.000	0.000	0.045	**1.000**
	**T3**	**A**	**1.000**	0.037	0.000	0.000	**1.000**	0.026	0.000	0.000		0.002	0.000	0.000	**0.883**	0.119	0.000	0.000	**0.799**	0.011	0.000	0.000
		**B**	0.000	**1.000**	0.078	0.000	0.000	**1.000**	0.007	0.000	0.006		0.000	0.000	0.240	**0.990**	0.000	0.000	0.342	**1.000**	0.000	0.000
		**C**	0.000	0.000	**0.967**	0.025	0.000	0.000	**1.000**	0.000	0.000	0.000		0.002	0.000	0.000	**1.000**	0.016	0.000	0.000	**1.000**	0.113
		**D**	0.000	0.000	0.002	**1.000**	0.000	0.000	0.031	**0.112**	0.000	0.000	0.332		0.000	0.000	0.000	**1.000**	0.000	0.000	0.052	**1.000**
	**T4**	**A**	**0.865**	0.332	0.000	0.000	**0.943**	0.247	0.000	0.000	**0.991**	0.062	0.000	0.000		0.994	0.000	0.000	**1.000**	0.813	0.000	0.000
		**B**	0.113	**0.940**	0.000	0.000	0.187	**0.933**	0.000	0.000	0.558	**0.579**	0.000	0.000	0.999		0.000	0.000	0.999	**1.000**	0.000	0.000
		**C**	0.000	0.007	**1.000**	0.000	0.000	0.012	**1.000**	0.000	0.000	0.065	**0.978**	0.003	0.000	0.000		0.001	0.000	0.000	**0.992**	0.009
		**D**	0.000	0.000	0.000	**1.000**	0.000	0.000	0.000	**0.989**	0.000	0.000	0.003	**0.958**	0.000	0.000	0.000		0.000	0.000	0.077	**1.000**
	**BW**	**A**	**0.015**	1.000	0.001	0.000	**0.029**	1.000	0.000	0.000	**0.217**	0.987	0.000	0.000	**0.899**	1.000	0.000	0.000		0.893	0.000	0.000
		**B**	0.013	**1.000**	0.001	0.000	0.025	**1.000**	0.000	0.000	0.201	**0.975**	0.000	0.000	0.880	**1.000**	0.000	0.000	1.000		0.000	0.000
		**C**	0.000	0.011	**1.000**	0.082	0.000	0.016	**1.000**	0.001	0.000	0.060	**1.000**	0.450	0.000	0.000	**1.000**	0.020	0.001	0.001		0.314
		**D**	0.000	0.000	0.042	**0.999**	0.000	0.000	0.252	**0.186**	0.000	0.000	0.816	**1.000**	0.000	0.000	0.050	**0.939**	0.000	0.000	0.704	

Ω_a			*Lophelia pertusa*																			
			**T1**				**T2**				**T3**				**T4**				**BW**			
			**A**	**B**	**C**	**D**	**A**	**B**	**C**	**D**	**A**	**B**	**C**	**D**	**A**	**B**	**C**	**D**	**A**	**B**	**C**	**D**
Madrepora oculata	**T1**	**A**		0.000	0.000	0.000	**0.992**	0.000	0.000	0.000	**1.000**	0.002	0.000	0.000	**0.833**	0.159	0.000	0.000	**0.920**	0.076	0.000	0.000
		**B**	0.001		0.042	0.000	0.052	**1.000**	0.002	0.000	0.004	**1.000**	0.000	0.000	0.227	**0.906**	0.018	0.000	0.146	**1.000**	0.003	0.000
		**C**	0.000	0.067		0.230	0.000	0.306	**1.000**	0.009	0.000	0.010	**0.980**	0.007	0.000	0.000	**1.000**	0.001	0.000	0.004	**0.999**	0.530
		**D**	0.000	0.000	0.019		0.000	0.000	0.833	**1.000**	0.000	0.000	0.997	**0.999**	0.000	0.000	0.394	**0.946**	0.000	0.000	0.994	**1.000**
	**T2**	**A**	**1.000**	0.005	0.000	0.000		0.004	0.000	0.000	**1.000**	0.168	0.000	0.000	**1.000**	0.974	0.000	0.000	**1.000**	0.816	0.000	0.000
		**B**	0.000	**1.000**	0.109	0.000	0.003		0.030	0.000	0.000	**0.999**	0.003	0.000	0.027	**0.388**	0.168	0.000	0.015	**0.943**	0.029	0.001
		**C**	0.000	0.008	**1.000**	0.127	0.000	0.015		0.131	0.000	0.001	**1.000**	0.105	0.000	0.000	**1.000**	0.021	0.000	0.000	**1.000**	0.941
		**D**	0.000	0.000	0.000	**0.998**	0.000	0.000	0.003		0.000	0.000	0.526	**1.000**	0.000	0.000	0.022	**1.000**	0.000	0.000	0.563	**1.000**
	**T3**	**A**	**1.000**	0.182	0.000	0.000	**1.000**	0.130	0.000	0.000		0.016	0.000	0.000	**0.970**	0.436	0.000	0.000	**0.990**	0.118	0.000	0.000
		**B**	0.000	**0.773**	0.979	0.000	0.000	**0.941**	0.700	0.000	0.002		0.000	0.000	0.512	**0.993**	0.004	0.000	0.375	**1.000**	0.001	0.000
		**C**	0.000	0.000	**0.990**	0.638	0.000	0.001	**1.000**	0.050	0.000	0.157		0.461	0.000	0.000	**0.998**	0.153	0.000	0.000	**1.000**	0.999
		**D**	0.000	0.000	0.101	**1.000**	0.000	0.000	0.426	**0.902**	0.000	0.000	0.945		0.000	0.000	0.017	**1.000**	0.000	0.000	0.504	**1.000**
	**T4**	**A**	**0.651**	0.552	0.000	0.000	**0.951**	0.419	0.000	0.000	**1.000**	0.004	0.000	0.000		1.000	0.000	0.000	**1.000**	0.984	0.000	0.000
		**B**	0.093	**0.954**	0.001	0.000	0.347	**0.936**	0.000	0.000	0.905	**0.015**	0.000	0.000	1.000		0.000	0.000	0.999	**1.000**	0.000	0.000
		**C**	0.000	0.038	**1.000**	0.034	0.000	0.065	**1.000**	0.001	0.000	0.941	**0.998**	0.162	0.000	0.000		0.003	0.000	0.002	**1.000**	0.689
		**D**	0.000	0.000	0.005	**1.000**	0.000	0.000	0.042	**1.000**	0.000	0.000	0.351	**1.000**	0.000	0.000	0.009		0.000	0.000	0.201	**1.000**
	**BW**	**A**	**0.022**	1.000	0.002	0.000	**0.116**	1.000	0.000	0.000	**0.687**	0.305	0.000	0.000	**0.992**	1.000	0.001	0.000		0.955	0.000	0.000
		**B**	0.028	**1.000**	0.002	0.000	0.143	**0.997**	0.000	0.000	0.733	**0.135**	0.000	0.000	0.996	**1.000**	0.001	0.000	1.000		0.000	0.000
		**C**	0.000	0.174	**1.000**	0.469	0.000	0.239	**1.000**	0.057	0.000	0.972	**1.000**	0.786	0.000	0.005	**1.000**	0.268	0.019	0.016		0.994
		**D**	0.000	0.000	0.823	**0.997**	0.000	0.000	0.991	**0.580**	0.000	0.053	1.000	**1.000**	0.000	0.000	0.904	**0.961**	0.000	0.000	0.996	
