# Supplementary material for: End of the Century pCO2 Levels Do Not Impact Calcification in Mediterranean Cold-Water Corals
Source: PLoS One. 2013 Apr 30;8(4):e62655. doi: 10.1371/journal.pone.0062655 (PMC3640017; doi:10.1371/journal.pone.0062655)
Supplement: Table S3 — Average calcification rates (G) of the cold-water corals Lophelia pertusa (LP) and Madrepora oculata (MO) at T0 to T4 determined by the total anomaly technique in monthly time intervals and 2 days of incubation, and calcification rates determined by buoyant weight (G(BW)) after maintenance of corals for 9–10 months under respective pCO2 treatment levels. (PDF) [file pone.0062655.s005.pdf]

**Table S3** Average calcification rates (G) of the cold-water corals *Lophelia pertusa* (LP) and *Madrepora oculata* (MO) at $T_0$ to $T_4$ determined by the total anomaly technique in monthly time intervals and 2 days of incubation, and calcification rates determined by buoyant weight (G(BW)) after maintenance of corals for 9-10 months under respective $pCO_2$ treatment levels.

| $pCO_2$ level | Coral | N | G ($T_0$) [%$d^{-1}$] | ± S.D. | G ($T_1$) [%$d^{-1}$] | ± S.D. | G ($T_2$) [%$d^{-1}$] | ± S.D. | G ($T_3$) [%$d^{-1}$] | ± S.D. | G ($T_4$) [%$d^{-1}$] | ± S.D. | G (BW) [%$d^{-1}$] | ± S.D. |
|---|---|---|---|---|---|---|---|---|---|---|---|---|---|---|
| A | LP | 4 | 0.005 | ± 0.003 | 0.004 | ± 0.001 | 0.014 | ± 0.013 | 0.017 | ± 0.019 | 0.007 | ± 0.002 | 0.010 | ± 0.008 |
| B | LP | 4 | 0.012 | ± 0.009 | 0.018 | ± 0.023 | 0.019 | ± 0.021 | 0.013 | ± 0.012 | 0.011 | ± 0.012 | 0.018 | ± 0.020 |
| C | LP | 5 | 0.004 | ± 0.004 | 0.009 | ± 0.008 | 0.013 | ± 0.015 | 0.017 | ± 0.018 | 0.004 | ± 0.002 | 0.013 | ± 0.020 |
| D | LP | 5 | 0.004 | ± 0.005 | 0.006 | ± 0.004 | 0.014 | ± 0.008 | 0.023 | ± 0.020 | 0.024 | ± 0.022 | 0.021 | ± 0.037 |
| A | MO | 4 | 0.017 | ± 0.004 | 0.015 | ± 0.005 | 0.029 | ± 0.016 | 0.028 | ± 0.018 | 0.020 | ± 0.015 | 0.017 | ± 0.014 |
| B | MO | 7 | 0.034 | ± 0.033 | 0.023 | ± 0.011 | 0.032 | ± 0.022 | 0.058 | ± 0.057 | 0.027 | ± 0.016 | 0.034 | ± 0.036 |
| C | MO | 6 | 0.014 | ± 0.015 | 0.022 | ± 0.010 | 0.022 | ± 0.011 | 0.029 | ± 0.016 | 0.020 | ± 0.013 | 0.025 | ± 0.017 |
| D | MO | 6 | 0.024 | ± 0.015 | 0.027 | ± 0.022 | 0.029 | ± 0.021 | 0.026 | ± 0.019 | 0.040 | ± 0.033 | 0.038 | ± 0.057 |
