# Supplementary material for: End of the Century pCO2 Levels Do Not Impact Calcification in Mediterranean Cold-Water Corals
Source: PLoS One. 2013 Apr 30;8(4):e62655. doi: 10.1371/journal.pone.0062655 (PMC3640017; doi:10.1371/journal.pone.0062655)
Supplement: Table S4 — Statistical results for repeated measures ANOVA of calcification rates (G). A Comparison between total alkalinity (TA) method (average G, pooled T1–T4) and Buoyant Weighting (BW) for pCO2 treatments A–D; and B for comparison of repeated measurements T0–T4 for the 4 pCO2 treatments. Table C gives the matrix for p-values of the Tukey-Honest-Significance post-hoc comparison for unequal N of the variable R1 (T0–T4) for M. oculata (lower left) and L. pertusa (upper right). Significant p are marked in bold, italic (PDF) [file pone.0062655.s006.pdf]

Table S4 Statistical results for repeated measures ANOVA of calcification rates (G). **A** Comparison between total alkalinity (TA) method (average G, pooled T_1-T_4) and Buoyant Weighting (BW) for pCO_2 treatments A-D; and **B** for comparison of repeated measurements T0-T4 for the 4 pCO2 treatments. Table **C** gives the matrix for p-values of the Tukey-Honest-Significance post-hoc comparison for unequal N of the variable R1 (T_0-T_4) for *M. oculata* (lower left) and *L. pertusa (upper right).* Significant p are marked in bold, italic

**A**	***Lophelia pertusa***					***Madrepora oculata***				
	SS	DF	MS	F	p	SS	DF	MS	F	p
**pCO2**	0.000	3	0.000	0.254	0.0857	0.002	3	0.000	0.397	0.757
**R1 (TA vs BW)**	0.000	1	0.000	0.258	0.619	0.000	1	0.000	0.004	0.951
**R1 * pCO2**	0.000	3	0.000	0.069	0.975	0.000	3	0.000	0.341	0.796

**B**	***Lophelia pertusa***					***Madrepora oculata***				
	SS	DF	MS	F	p	SS	DF	MS	F	p
**pCO2**	0.001	3	0.000	0.345	0.793	0.004	3	0.001	0.684	0.573
**R1 (T0-T4)**	0.001	4	0.000	4.506	***0.003***	0.003	4	0.001	3.077	***0.021***
**R1 * pCO2**	0.002	12	0.000	1.658	0.102	0.004	12	0.000	1.714	0.080

C p-values for unequal N HSD; variable $\mathbf{T_0}$-$\mathbf{T_4}$
Approximate Probabilities for Post Hoc Tests.
Error: Within MS = .00008, df = 56.000 for *L. pertusa; Witin MS = 0.00022, df = 76 for* M. oculata

		Lophelia pertusa					
	**G [%d⁻¹]**	**0.006**	**0.009**	**0.015**	**0.018**	**0.012**	
		To	T1	T2	T3	T4	
Madrepora oculata	T0		0.777	***0.029***	***0.002***	0.259	***Lophelia pertusa***
	T1	1.000		0.341	***0.046***	0.900	
	T2	0.812	0.708		0.867	0.860	
	T3	***0.018***	***0.010***	0.234		0.299	
	T4	0.865	0.773	1.000	0.189		
		To	T1	T2	T3	T4	
	**G [%d⁻¹]**	**0.023**	**0.023**	**0.028**	**0.037**	**0.028**	
		Madrepora oculata					
