# Supplementary material for: End of the Century pCO2 Levels Do Not Impact Calcification in Mediterranean Cold-Water Corals
Source: PLoS One. 2013 Apr 30;8(4):e62655. doi: 10.1371/journal.pone.0062655 (PMC3640017; doi:10.1371/journal.pone.0062655)
Supplement: File S1 — Method comparison between total alkalinity anomaly (TAA) and buoyant weight (BW) technique to establish net calcification rates (G) of M. oculata and L. pertusa . (PDF) [file pone.0062655.s007.pdf]

SI 1 Method comparison between total alkalinity anomaly (TAA) and buoyant weight (BW) technique to establish net calcification rates (G) of *M. oculata* and *L. pertusa*

A comparison between two methods to establish net calcification rates (G) of *M. oculata* and *L. pertusa* by the total alkalinity anomaly (TAA; Chisholm & Gattuso, Limnol. Oceanogr. 36: 1232-1239, 1991) and buoyant weight (BW; Davies: Mar. Biol. 101: 389-395, 1989) technique has been conducted for measurements carried out over a similar time interval (123 and 137 days, respectively). G established by the TAA method reflect distinct growth increments (here measured during 2-day incubation), while the BW method is the integral growth over time as it requires that G is measured over a longer time period to be able to detect measurable changes in $CaCO_3$ accretion. It is therefore problematic to compare G determined by a single discrete TAA measurement at a given time with that of BW. Here, we can compare 5 growth increments derived from TAA (T_0-T_4) during a similar time range (123 days) as that established by BW (137 days). Original data for G and coral fragments (ID) used for comparison are given in Table $SI_{TAAvsBW}$. Paired t-test for TAA pooled over the 5 growth increments (T_0-T_4) and the BW method revealed that the methods provide comparable results with respect to G (Table SI T-test$_{TAAvsBW}$) for both cold-water coral species *M. oculata* and *L. pertusa* studied.

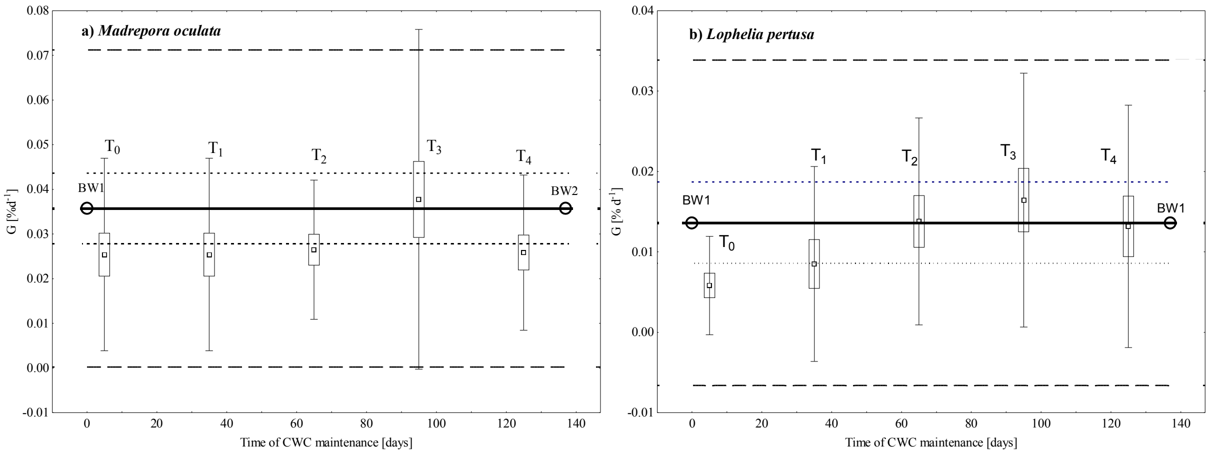

Fig. $SI_{TAAvsBW}$ Comparison of the buoyant weight (BW) and total alkalinity anomaly technique (TAA, T_0-T_4) to establish net calcification rates of the cold-water corals **a)** *M. oculata* (N=20) and **b)** *L. pertusa* (N=16). BW comprises the integral growth determined over a periods of 137 days, while the TAA method is derived from 2-days incubation and represents therefore discrete growth rates at the time of measurement of the same cold-water coral fragments (see Table $SI_{TAAvsBW}$) used for comparison of the 2 methods. TAA (T_0-T_4) are box-whisker plots with mean, S.E. and S.D., BW is given as mean for integral growth (line) between time when BW was established (BW1-BW2), S.E. (pointed line) and S.D. (plotted line) for the whole time range.

Table $SI_{TAAvsBW}$ Comparison of total alkalinity anomaly for single 2-day growth increments (T_0-T_4) and pooled for the T_0-T_4 (TAA) and bouyant weight (BW) technique to establish net calcification rates (G) of *Madrepora oculata* and *Lophelia pertusa*. Data are G [% d^{-1}], ID gives the respective coral fragment used for comparison.

Madropora oculata								*Lophelia pertusa*							
ID	To	T1	T2	T3	T4	TAA	BW	ID	To	T1	T2	T3	T4	TAA	BW
A18	0.021	0.018	0.019	0.017	0.008	0.017	0.014	A10	0.006	0.004	0.005	0.005	0.005	0.005	0.003
A19	0.013	0.008	0.010	0.009	0.008	0.010	0.003	A16	0.008	0.005	0.033	0.045	0.010	0.020	0.029
A7	0.015	0.020	0.045	0.036	0.039	0.031	0.040	A2	0.005	0.004	0.008	0.006	0.007	0.006	0.005
B10	0.064	0.030	0.037	0.048	0.037	0.043	0.100	A4	0.000	0.004	0.012	0.013	0.006	0.007	0.007
B18	0.014	0.008	0.003	0.010	0.009	0.009	0.005	B16	0.005	0.003	0.006	0.003	0.007	0.005	0.002
B19	-0.001	0.011	0.009	0.053	0.021	0.019	0.008	B2	0.025	0.052	0.051	0.030	0.028	0.037	0.040
B6	0.019	0.023	0.040	0.066	0.021	0.034	0.038	B3	0.006	0.008	0.008	0.008	0.010	0.008	0.004
B7	0.019	0.019	0.026	0.025	0.024	0.023	0.024	C1	0.006	0.009	0.016	0.025	0.003	0.012	0.009
B8	0.093	0.040	0.065	0.181	0.059	0.088	0.133	C16	0.001	0.002	0.005	0.005	0.001	0.003	0.003
B9	0.031	0.027	0.046	0.025	0.022	0.030	0.027	C2	0.010	0.010	0.008	0.008	0.005	0.008	0.003
C19	0.030	0.035	0.031	0.038	0.026	0.032	0.020	C4	0.001	0.002	0.000	0.002	0.005	0.002	0.005
C6	0.024	0.034	0.033	0.049	0.040	0.036	0.046	D1	0.012	0.012	0.022	0.054	0.062	0.032	0.078
C7	0.014	0.018	0.016	0.013	0.020	0.016	0.025	D16	0.005	0.004	0.011	0.003	0.008	0.006	0.008
C8	0.015	0.015	0.025	0.040	0.023	0.024	0.070	D2	0.001	0.006	0.013	0.014	0.026	0.012	0.002
C9	0.017	0.020	0.023	0.028	0.008	0.019	0.026	D3	0.001	0.008	0.021	0.015	0.010	0.011	0.017
D10	0.019	0.022	0.030	0.007	0.029	0.021	0.019	D4	0.001	0.002	0.002	0.028	0.017	0.010	0.003
D18	0.018	0.014	0.014	0.013	0.013	0.014	0.010								
D19	0.007	0.007	0.012	0.009	0.005	0.008	0.010								
D7	0.023	0.020	0.034	0.035	0.034	0.029	0.008								
D8	0.052	0.069	0.068	0.053	0.072	0.063	0.088								

Table paired T-Test$_{TAAvsBW}$ Mean, S.D.and S.E. of calcification rates (G) of *M. oculata (MO)* and *L. pertusa (LP)* for total alkalinity anomaly (TAA, data pooled T_0-T_4) and bouyant weight method and results of paired t-test between TAA and BW method.

Coral	Method	GMean [%d^{-1}]	S.D.	S.E.	N	Diff.	S.D. Diff.	t	df	p
MO	TAA_{T0-4}	0.028	0.019	0.004						
MO	BW	0.036	0.035	0.008	20	-0.007	0.021	-1.605	19	0.125
LP	TAA_{T0-4}	0.012	0.010	0.002						
LP	BW	0.014	0.020	0.005	16	-0.002	0.013	-0.673	15	0.511
